# From tumor targeting to tumor accessibility: surface-engineered bacteria as precision living therapeutics for translational medicine

**DOI:** 10.1186/s13045-026-01827-1

**Published:** 2026-07-07

**Authors:** Liyuan Qiao, Leyang Wu, Yihan Xiao, Jiahui Qiu, Xinyue Qiao, Xiao Dong, Chengxuan Lei, Wanru Wang, Yuzi Zhao, Zichun Hua

**Affiliations:** 1 State Key Laboratory of Pharmaceutical Biotechnology, School of Life Sciences, Nanjing University, Nanjing, 210023 Jiangsu P.R. China; 2 Nanjing Generecom Biotechnology Co., Ltd, Nanjing, 210023 Jiangsu P. R. China; 3 Changzhou High-Tech Research Institute of Nanjing University and Jiangsu TargetPharma Laboratories, Inc, Changzhou, 213164 Jiangsu P. R. China; 4 Faculty of Pharmaceutical Sciences, Henan Medical University, Xinxiang, 453002 Henan P. R. China

**Keywords:** Bacterial therapeutics, Surface modification, Bacterial clinical translation, Cancer immunotherapy, Cellular camouflage

## Abstract

The concept of bacterial therapy dates back over a century to clinical observations that incidental infections could induce tumor regression. Recent advances in genetic engineering and synthetic biology have since transformed bacteria into versatile living therapeutics with significant preclinical potential against diseases such as cancer, inflammatory disorders, and metabolic conditions. However, clinical translation faces considerable hurdles. Here, we provide a clinically oriented perspective on the translational gap in bacterial therapy. Drawing inspiration from the success of antibody-drug conjugates in achieving precise payload delivery, we highlight an emerging paradigm of “precision living therapeutics” enabled by bacterial surface engineering. we propose the concept of “Tumor accessibility” for the first time, and identify its insufficiency as a critical bottleneck in current therapeutic applications. We then systematically summarize recent advances in bacterial surface engineering, encompassing physical, chemical, and biological strategies, with a focus on their capacity to evade immune clearance, enhance tumor colonization, and improve therapeutic performance. Chemical approaches primarily involve covalent conjugation, including the SpyTag/SpyCatcher system and bioorthogonal click chemistry-based metabolic labeling. Physical strategies center on cell membrane encapsulation and surface coatings such as layer-by-layer encapsulation. Biological strategies include cell camouflage and genetic modulation of surface structures, display of functional biomolecules, and affinity-based systems such as biotin-streptavidin interactions. Finally, we discuss integrative strategies that combine surface-engineered bacteria with conventional treatment modalities, including physical therapy, chemotherapy, and immunotherapy. We propose that future clinical translation of bacterial therapy should shift from localized modification design to a holistic consideration of systemic accessibility.

## Introduction

Cancer therapy has undergone profound transformation over the past century, progressing from radiotherapy and chemotherapy to molecularly targeted agents and cell-based precision interventions. Radiotherapy and chemotherapy have substantially improved survival across many malignancies, yet their clinical utility is often constrained by systemic toxicity and limited tumor selectivity, particularly in the context of intratumoral heterogeneity. The emergence of precision medicine has led to the development of antibody‑drug conjugates (ADCs) [1, 2] and other drug conjugate (XDC) platforms, and engineered cell therapies such as chimeric antigen receptor (CAR) T therapy [3, 4], which combine targeting specificity with potent effector functions. In solid tumors, however, therapeutic performance remains influenced by insufficient tissue penetration [5, 6], heterogeneous antigen expression [7, 8], and toxicity, underscoring the persistent importance of effective in vivo delivery.

As a living therapeutic agent, the intrinsic tropism of bacteria for hypoxic and necrotic tumor regions, together with active motility and the capacity for in situ proliferation, enables accumulation within tumor cores that are often inaccessible to conventional drugs. Advances in genetic engineering and synthetic biology have enabled attenuation of virulence and precise programming of therapeutic functions [9, 10], allowing engineered strains to secrete immunomodulatory factors [11, 12], cytotoxic proteins [13–15], or nucleic acids [16, 17] in a spatially restricted manner. Beyond oncology, engineered bacteria have also been explored for metabolic and inflammatory diseases [18, 19], highlighting their versatility as therapeutic platforms.

**Fig. 1:**
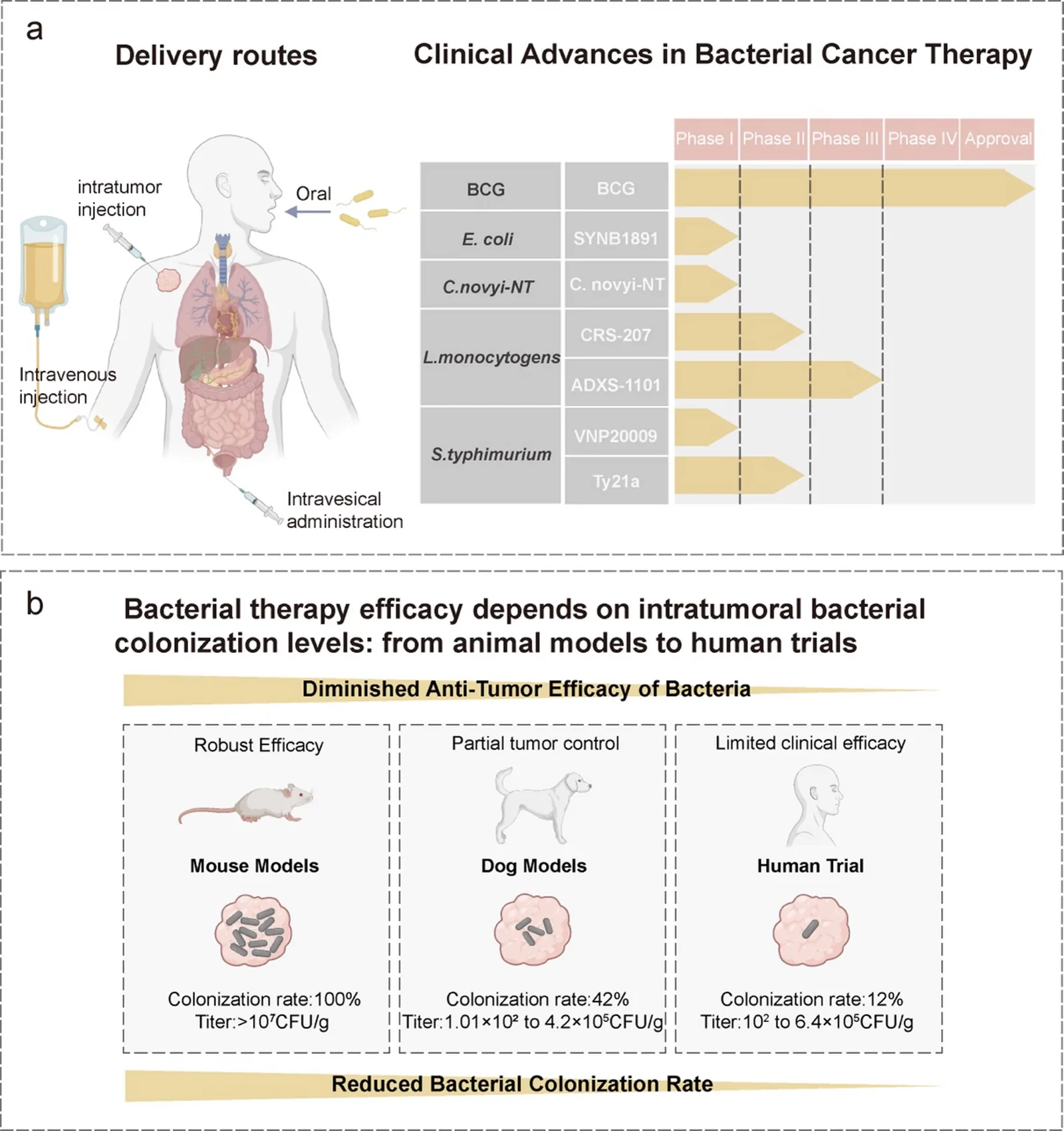
Clinical progress of bacterial-mediated antitumor therapy. **(a)** Primary routes of administration for bacterial antitumor therapy and the current clinical progress of different bacterial species.(**b**) Colonization levels and antitumor efficacy of intravenously administered bacteria in mouse and dog models, as well as in human clinical trials, with Salmonella VNP20009 as the representative strain. Colonization is defined by two complementary metrics: colonization rate (the percentage of subjects with detectable intratumoral bacteria in a given group) and colonization titer (the intratumoral bacterial titer (reported as CFU/g) quantified from subjects with detectable colonization).The titer data were collected at the following time points post-bacterial administration: days 3–5 for mice [22–24], day 7 for dogs [26], and days 2–4 for humans [28]. Direct quantitative comparisons across species are subject to cross-species heterogeneity in tumor models, dosing regimens, and sampling time points

Due to safety and efficacy concerns, the clinical translation of bacterial cancer therapy remains limited. With the exception of Bacillus Calmette-Guérin, an attenuated strain of *Mycobacterium bovis* approved for high-risk non-muscle-invasive bladder cancer [20, 21], most bacterial therapeutics remain in preclinical or early-phase development **(**Fig. 1a**)**. A central limitation lies in the insufficient accessibility of systemically administered bacteria to tumor tissues **(**Fig. 1b**)**, which constrains their capacity to exert consistent therapeutic effects. In recent years, surface engineering has emerged as a genome-independent strategy to enhance bacterial in vivo delivery efficiency and therapeutic effect.

In this review, we summarize the evolution of bacterial cancer therapy and analyze key translational bottlenecks, particularly insufficient bacterial colonization at disease sites, while highlighting surface engineering as a promising solution. We then review physical, chemical and biological modification strategies for engineering bacterial surfaces and discuss their applications across different disease contexts, such as tumors and inflammatory bowel disease. Finally, using cancer therapy as a case study, we illustrate the synergistic potential of surface-engineered bacteria when integrated with physical, chemical, or immunotherapeutic modalities.

## Development and clinical potential of bacterial therapy

In recent years, research on engineered bacterial therapies has accelerated, demonstrating remarkable potential not only in cancer but also across a broad spectrum of diseases. This rapid progress has been fueled by deeper insights into bacterial targeting mechanisms and, critically, by the development of attenuated strains with significantly improved safety. These advances have expanded the applicability of engineered bacteria to oncology, immunometabolic disorders, infectious diseases, and regulation of gut homeostasis.

Early bacterial strains used in research, for example, Serratia marcescens in Coley’s toxin—were highly pathogenic and, according to the Biosafety in Microbiological and Biomedical Laboratories (BMBL) 6th Edition, typically required handling at biosafety level 2 (BSL-2) or higher **(**Table 1**)**. BSL-2 laboratories mandate biological safety cabinets or equivalent containment, rigorous waste-decontamination procedures, and detailed emergency protocols for accidental exposure. These strict requirements impose substantial facility and operational burdens, limiting the number of research teams capable of working with such pathogens and consequently restricting both the scope and depth of investigations. In contrast, attenuated engineered strains, such as the *Salmonella typhimurium* VNP20009 (with deletions in *purI* and *msbB*), exhibit markedly reduced virulence through systematic genetic attenuation. VNP20009 has no formal biosafety classification from regulatory authorities **(**Table 1**)**, and its safety profile has been extensively evaluated in multiple clinical trials for cancer therapy, where it demonstrated acceptable tolerability in patients. These clinical data, together with accumulated laboratory safety records, indicate that VNP20009 poses handling risks comparable to standard BSL-1 strains such as *E. coli* Nissle 1917(EcN). All handling of VNP20009 should follow institutional biosafety review and risk assessment. BSL-1 laboratories require only basic microbiological practices and typically do not require biosafety cabinets for routine manipulation. This effective transition from BSL-2 to quasi-BSL-1 conditions significantly lowers research barriers, enhances biosafety, and enables broader preclinical evaluation across diverse animal models. In various mouse tumor models, the titers of VNP20009 at tumor sites following intravenous administration typically range from 10⁷ to 10⁹ CFU/g at 3–5 days post-injection [22–24]. In a rabbit VX2 liver tumor model, a single intravenous injection of *Clostridium novyi-NT* (*C. novyi-NT*) spores led to tumor-specific germination and extensive intratumoral necrosis, with no germination in normal tissues. Correspondingly, 7 of 23 rabbits (30%) achieved complete radiologic remission, demonstrating that robust intratumoral colonization is sufficient to induce profound tumor destruction and, in some cases, complete regression [25]. Similarly, in a clinical study involving 41 pet dogs with spontaneous malignant tumors, systemic administration of VNP20009 (1.5 × 10⁵−1 × 10⁸ CFU/kg) yielded a maximum tolerated dose of 3 × 10⁷ CFU/kg. One week post-treatment, bacterial presence was detected in expanded tumor cultures from 42% of dogs, with 21% showing quantifiable intratumoral burdens (1.01 × 10²−4.2 × 10⁵ CFU/g). These colonization profiles corresponded with meaningful clinical responses—10% complete response, 5% partial response, and 10% stable disease—for an overall clinical benefit rate of 25% [26]. In summary, bacterial therapy has demonstrated significant anti-tumor potential in a variety of preclinical animal models, providing robust proof-of-concept for the efficacy of engineered bacteria and directly advancing their translation toward clinical application.

**Table 1:** Required laboratory biosafety levels for different bacterial strains

Bacterial strain	Biosafety level (BSL)
*Streptococcus pyogenes*	BSL-2
*Salmonella Serotypes*	BSL-2
*Listeria monocytogenes*	BSL-2
*Clostridium*	BSL-2
*Escherichia coli* (non-pathogenic strains)	BSL-1
VNP20009	NA* (Safety profile comparable to BSL-1; not formally classified)

Biosafety levels are from Biosafety in Microbiological and Biomedical Laboratories (6th Edition)

*For VNP20009, NA indicates that no formal biosafety classification has been assigned by regulatory authorities; its safety profile is comparable to BSL-1 based on accumulated evidence, but this does not constitute an official classification. All handling should follow institutional biosafety review and risk assessment

## Insufficient bacterial accumulation at disease sites is the key barrier to clinical translation

In the transition from preclinical research to human application, this therapeutic approach faces multiple challenges. The inherent heterogeneity of the human tumor microenvironment, which is characterized by immunosuppressive cell infiltration, a dense fibrotic stroma, aberrant vascular architecture, and unique hemodynamic features [5, 27], precludes the direct extrapolation of efficacy data obtained from animal models to clinical outcomes. In light of these challenges, a series of clinical trials have been undertaken to systematically evaluate the safety, tolerability, and preliminary efficacy of this therapeutic approach in humans. Only a limited number of these trials have incorporated the assessment of intratumoral bacterial colonization **(**Table 2**)**.

**Table 2:** Clinical applications of bacterial therapies

Biological/ tradename	Background strain	Indications	Trail	Engineered strain	Combination therapy	Dose	Administration	Completion	Bacterial colonization in lesion	Phase	Status	*n*
VNP20009	*S. typhimurium* (VNP20009)	Advanced or metastatic cancer	NCT00004988	None	None	NR	Intravenous	2002	NR	I	Completed	45
VNP20009	*S. typhimurium* (VNP20009)	Metastatic melanoma and metastatic renal cell carcinoma	Toso et al., 2002	None	None	Dose escalation: 10^6^ to 10^9^ CFU/m² BSA	Intravenous	2002	Colonization in 12% (3/25) of patients, with day 2–4 titers ranged from 10²−6.4 × 10⁵ cfu/g	I	Completed	25
VNP20009	S. typhimurium (VNP20009)	Metastatic melanoma	David et al.,2003	None	None	3 × 10^8^CFU/m² BSA	Intravenous	2003	Colonization in 1/4 patients (25%); detected as a single colony	I	Completed	4
VNP20009	*S. typhimurium* (VNP20009)	Advanced or metastatic solid tumors	NCT00004216	None	None	NR	Intratumoral	2008	NR	I	Completed	NR
x4550	*S. typhimurium* (virulent strain x4550)	Solid tumors with unresectable hepatic metastases	NCT01099631	Engineered to express human IL-2	None	Dose escalation: 1 × 10^5^ to 1 × 10^10^ CFU	Oral	2014	NR	I	Completed	22
Saltikva	an attenuated strain of *S. typhimurium*	metastatic pancreatic cancer	NCT04589234	Engineered to express human IL-2	FOLFIRINOX or Gemcitabine/Abraxane	Multi-dose, 1 × 10⁹ CFU/dose	Oral	ongoing	NR	II	Active, not recruiting	60
VNP20009	*S. typhimurium* (VNP20009)	advanced solid tumors	NCT00006254	None	None	NR	Intravenous	2008	NR	I	Completed	NR
TAPET-CD	*S. typhimurium* (VNP20009)	Refractory solid tumors	John et al., 2003	Engineered to express CD	5-Fluorocytosine (5-FC)	Dose escalation: 3 × 10^6^ to 3 × 10^7^ CFU/m^2^	Intratumoral	2008	Colonization in 2/3 patients; persistence ≥ 15 days (Day 15 titer range: 84 − 2.6 × 10⁵ CFU/g)	I	Completed	3
Ty21a/Vivotif^®^	*S. Typhi* (Ty21a)	non-muscle invasive bladder cancer	NCT03421236	None	None	Multi-dose, 1 × 10^8^ CFU	Intravesical	2022	NR	I	Unknown status	25
VXM01	*S. Typhi* (Ty21a)	progressive glioblastoma	NCT03750071	Engineered to express eukaryotic VEGFR-2	Avelumab	NR	NR	2021	NR	I/II	Unknown status	30
SYNB1891	*E. coli* Nissle 1917 (EcN)	advanced/metastatic solid tumor or lymphoma	NCT04167137	Engineered to express STING Agonist	Monotherapy or in combination with atezolizumab	Dose escalation: 1 × 10^6^ to 3 × 10^8^ CFU or 1 × 10^7^ to 3 × 10^7^ CFU with Atezolizumab	Intratumoral	2021	Not detected in tumor at Day 7 post-dose.	I	Terminated	24
Mutaflor^®^	*E.coli* Nissle 1917 (EcN)	Mild-to-moderate ulcerative colitis	NCT04969679	None	5-aminosalicylic acid (5-ASA)	Multi-dose, 1–2 capsules	Oral	2020	NR	IV	Completed	134
SYNB1020CP001	*E.coli* Nissle 1917 (EcN)	Urea Cycle Disorder	NCT03179878	Engineered to convert ammonia to L-arginine	None	Multi-capsule dose, 2.5 × 10⁹–2.5 × 10¹⁰ CFU/capsule	Oral	2018	NR	I	Completed	52
*C. novyi-NT* spores	Attenuated *C. novyi-NT*, an α-toxin-deficient, nontoxic strain.	refractory solid tumor malignancies	NCT01924689	None	None	Dose escalation: 1 × 10^4^ to 3 × 10^6^ spores/kg	Intratumoral	2017	Germination in 42% (10/24) of patients.	I	Completed	24
*C. novyi-NT* spores	Attenuated *C. novyi-NT*, an α-toxin-deficient, nontoxic strain.	Treatment-refractory solid tumors	NCT00358397	None	None	Single dose	Intravenous	2008	NR	I	Terminated	2
*C. novyi-NT* spores	Attenuated *C. novyi-NT*, an α-toxin-deficient, nontoxic strain.	solid tumor malignancies	NCT01118819	None	None	Dose escalation: 1 × 10⁵ to 1 × 10⁷ spores/kg	Intravenous	2013	NR	I	Terminated	5
JNJ-64,041,757	*L. monocytogenes* LADD	advanced or metastatic NSCLC	NCT02592967	Engineered to express human mesothelin	None	Multi-dose, Dose escalation: 1 × 10^8^ to 1 × 10^9^ CFU	Intravenous	2018	NR	I	Terminated	18
JNJ-64,041,757	*L. monocytogenes* LADD	Advanced Adenocarcinoma of the Lung	NCT03371381	Engineered to express human mesothelin	Nivolumab	Multi-dose, 1 × 10^9^ CFU	Intravenous	2018	NR	Ib/II	Terminated	12
ADXS11-001	*L. monocytogenes* LADD	HPV associated OPSCC	NCT01598792	Engineered to express HPV-16	None	Dose escalation: 3.3 × 10^8^ to 3.3 × 10^9^ CFU	Intravenous	2014	NR	I	Terminated	2
ADXS11-001	*L. monocytogenes* LADD	Locally advanced cervical cancer	NCT02853604	Engineered to express tLLO-HPV-16 E7	None	Multi-dose,1 × 10^9^ CFU	Intravenous	2019	NR	III	Terminated	110
CRS-207	*L. monocytogenes* LADD	Advanced solid tumors	NCT00585845	Engineered to express human mesothelin.	None	Multi-dose, dose escalation: 1 × 10⁸ to 1 × 10¹⁰ CFU	Intravenous	2009	NR	I	Terminated	17
CRS-207	*L. monocytogenes* LADD	Metastatic pancreatic adenocarcinoma	NCT02004262	Engineered to express human mesothelin.	Monotherapy or in combination with Cy/GVAX	Multi-dose, 1 × 10^9^ CFU	Intravenous	2016	NR	IIb	Completed	303
ADXS31-142	Attenuated *L. monocytogenes*	Metastatic castration-resistant prostate cancer	NCT02325557	Engineered to secrete tLLO-PSA	Monotherapy or in combination with pembrolizumab	Multi-dose, dose escalation: 1 × 10⁹ to 1 × 10¹⁰ CFU, 1 × 10⁹ CFU with pembrolizumab	Intravenous	2021	NR	I/II	Completed	50
BCG	BCG	Bladder cancer	NCT02311101	None	Mitomycin C	Dose escalation: 2 × 10^8^ to 8 × 10^8^ CFU	Intravesical	2015	NR	I	Completed	18
BCG	BCG	Non-muscle invasive bladder cancer	NCT04165317	None	Monotherapy or in combination with Sasanlimab	NR	Intravesical	2024	NR	III	Active, not recruiting	1394
BCG	BCG	Non-muscle invasive bladder cancer	NCT03528694	None	Monotherapy or in combination with Durvalumab	Multi-dose, 1 × 10^8^−8 × 10^8^CFU with intravenous durvalumab	Intravesical	2025	NR	III	Active, not recruiting	1018

*S. typhimurium: Salmonella typhimurium; C. novyi-NT: Clostridium novyi-NT; L. monocytogens: Listeria monocytogens; E. coli: Escherichia coli; BCG: Bacillus Calmette-Guérin.*

NR: Not Reported; CFU: Colony-Forming Unit; BSA: Body-surface area; CD: Cytosine deaminase gene; VEGFR: Vascular endothelial growth factor receptor; STING: Stimulator of interferon genes; NSCLC: Non small cell lung cancer; HPV: Human papilloma virus; OPSCC: Oropharyngeal cancer; HPV-16: Human papilloma virus genotype 16 target antigens; tLLO: Truncated fragment of the listeriolysin toxin; PSA: Prostate-specific antigen; Cy: Cyclophosphamide, GVAX: Granulocyte-macrophage colony-stimulating factor-secreting allogeneic pancreatic tumor vaccine

This translational gap is starkly illustrated by the clinical experience with VNP20009, the first engineered *Salmonella* candidate evaluated in human oncology, was tested in a phase I trial involving 24 patients with metastatic melanoma and one with metastatic renal cell carcinoma. The maximum tolerated dose was 3 × 10⁸ CFU/m², while 10⁹ CFU/m² produced dose-limiting toxicities. Although colonization was detected at high doses, no objective tumor regression occurred, likely due to insufficient intratumoral bacterial accumulation [28]. A subsequent study using prolonged high-dose infusions (3 × 10⁸ CFU/m² over 4 h) showed rapid bloodstream clearance within six hours. In three of four patients, no bacteria were detected in tumor tissue after two weeks; even the single patient with low-level colonization exhibited no objective response [29]. These findings indicate that minimal colonization is inadequate for efficacy and may explain the limited success of intravenous administration.

The stark contrast between preclinical efficacy and clinical reality underscores a fundamental translational barrier: as host size and physiological complexity increase, intratumoral bacterial accumulation tends to decline, and this decline parallels the loss of therapeutic efficacy **(**Fig. 1b**)**. These observations, which account for the limited clinical responses observed to date, prompted us to introduce the concept of bacterial tumor accessibility. Defined as the ability of systemically delivered bacteria to survive immune surveillance, home to tumor sites, and penetrate the tumor microenvironment, this accessibility represents the sine qua non for bacterial antitumor effect. In preclinical models, researchers commonly use “targeting ability” to evaluate bacterial delivery efficiency. However, small animals such as mice exhibit short circulation times and relatively simple immune environments, which fail to adequately recapitulate the complex and sustained immune clearance processes in humans. Specifically, these limitations stem from several profound interspecies differences. Hemodynamically, mice exhibit significantly faster blood circulation driven by shorter vascular networks and a higher cardiac output-to-body weight ratio, humans circulate their total blood volume approximately 1–2 times per minute, whereas mice do so 3–14 times per minute [30]. Structurally, the abnormal and highly permeable tumor vasculature in mice allows bacteria to extravasate with relative ease [31]. Immunologically, rodents possess a substantially lower proportion of circulating neutrophils (15–20% versus 50–60% in humans [32]), which translates to diminished phagocytic clearance. Furthermore, murine complement-dependent bactericidal activity is fundamentally impaired. Unlike human serum, which rapidly orchestrates the lysis of Gram-negative bacteria (such as *Salmonella* [33] and *Escherichia coli* [34]) via efficient, C5b-dependent assembly of the membrane attack complex (MAC) [35], murine serum fails to mount a comparable response—a discrepancy likely rooted in divergent MAC assembly efficiencies. In the human body, even bacteria with ideal targeting capability face the risk of being eliminated by host defense systems before reaching the tumor. Tumor targeting, after all, utilizes affinity ligands on the surface to achieve specific homing to tumor tissues, increased retention at the tumor site, and uptake by tumor cells [36, 37], it answers “where to go”. This receptor-limited recognition event often suffices in the permissive murine tumor microenvironment, where immune pressure is low and vasculature is leaky. Tumor accessibility, by contrast, asks “whether and how to get there, survive, and colonize”—a multi-dimensional property integrating half-life, colonization rate, titer, and penetration depth within the tumor, which becomes the true bottleneck as immune and stromal barriers intensify from mice to humans [38, 39]. What works as tumor targeting in a mouse thus becomes, in a human, a question of tumor accessibility. Therefore, “tumor accessibility” is a more suitable concept at the clinical level.

Intravenous delivery is broadly applicable for small, diffuse, or metastatic lesions, but attenuated strains often struggle to balance biosafety and efficacy due to dose limitations. Consequently, direct intratumoral injection has been explored as an alternative, enabling substantially higher local bacterial concentrations while minimizing systemic exposure. In one study, VNP20009 engineered to express cytosine deaminase (CD) was administered intratumorally (3 × 10⁶–3 × 10⁷ CFU/m²) to patients with refractory solid tumors, combined with oral 5-fluorocytosine (5-FC). Two patients achieved persistent intratumoral colonization for ≥ 15 days, with confirmed prodrug conversion and a tumor-to-plasma 5‑fluorouracil (5-FU) ratio of 3:1; in contrast, the patient without colonization exhibited a ratio < 1.0. No injection-related adverse events were reported throughout the treatment cycles [40]. These results appear to highlight two points: (1) intratumorally delivered bacteria appear capable of achieving sustained local proliferation and high intratumoral titers; and (2) such high titers may contribute to therapeutic efficacy. Although precision-guided injection devices expand the applicability of intratumoral delivery to deep or otherwise inaccessible lesions [41], this approach remains constrained by tumor accessibility, lesion size, procedural complexity, safety concerns (e.g., tumor-wall disruption), cost, and limited convenience. Moreover, not all microbes are suitable for intratumoral use; for example, intratumoral administration of *ΔactA Listeria monocytogenes* has been shown to promote tumor growth by recruiting immunosuppressive neutrophils and establishing a microenvironment that supports immune evasion and long-term bacterial persistence [42].

Oral administration offers convenience for gastrointestinal diseases, but harsh gastric conditions substantially reduce bacterial viability. To overcome this challenge, microencapsulation [43, 44] has emerged as a key strategy in probiotic formulation. This technique involves the entrapment of bacterial cells within a protective matrix or wall material, significantly enhancing their survival during gastrointestinal transit. Clinically, probiotics are also combined with prebiotics [45–47] to improve gut conditions **(**Table 3**)**. SB-121—a formulation comprising *Lactobacillus reuteri*, Sephadex^®^ (dextran microparticles), and maltose—enhances adhesion, gastric survival, and persistence, and was shown to be safe and well tolerated in individuals with autism spectrum disorder [48], although detailed gastrointestinal assessments were lacking. Duolac Care, a dual-coated probiotic for irritable bowel syndrome, uses peptide and polysaccharide layers to protect *Lactobacillus* and *Bifidobacterium* from gastric degradation. Clinical studies demonstrated significant symptom improvement within two weeks and superior stool normalization compared with uncoated strains [49]. These findings highlight that surface coatings improve colonization and accelerate therapeutic onset by increasing local bacterial density at target sites, thereby mitigating delays caused by insufficient delivery.

**Table 3:** Clinical applications of surface-modified bacterial strains

Biological/ tradename	Bacterial Strain	Indications	Trail	Surface modification	Effect on modified bacteria	Dose	Administration	Completion year	*n*	Reference
SB-121	*Lactobacillus Reuteri*	ASD	NCT04944901	Co-formulation with glucose and maltose.	Improved epithelial adhesion and enhanced gastric survival.	Multi-dose, 2 × 10^10^ CFU	Oral	2023	15	[48]
Duolac Care	probiotic mixture	IBS	KCT0001226	Dual-layer Peptide-polysaccharide dual coating.	Increased viability under gastrointestinal acidity and bile exposure.	Multi-dose, 5 × 10^9^ CFU	Oral	2016	46	[49]
gQlab	probiotic capsule	IBS	NCT03964103	Quadruple coating with a water-soluble polymer, hyaluronic acid, porous particles, and protein.	Enhanced acid and bile tolerance, anti-inflammatory activity, and epithelial adhesion.	Multi-dose, 1 × 10^9^ CFU/strain	Oral	2018	109	[172]

ASD: Autism spectrum disorder; IBS: Irritable bowel syndrome

Probiotic mixture: *Lactobacillus acidophilus*, *Lactobacillus plantarum*, *Lactobacillus rhamnosus*, *Bifidobacterium longum*, *Bifidobacterium lactis*, and *Streptococcus thermophilus*

probiotic capsule: *Bifidobacterium longum* IDCC 4101, *Bifidobacterium bifidum* IDCC 4201, *Bifidobacterium lactis* IDCC 4301, *Bifidobacterium breve* IDCC 4401, *Enterococcus faecium* IDCC 2102, *Lactobacillus rhamnosus* IDCC 3201, *Lactobacillus acidophilus* IDCC 3302, *Lactobacillus casei* IDCC 3451, *Lactobacillus plantarum* IDCC 3501, and *Lactobacillus helveticus* IDCC 380

Collectively, these clinical observations underscore that the therapeutic efficacy of bacterial therapy is contingent upon bacterial localization at the pathological site. Notably, clinical trials have demonstrated that surface engineering strategies can markedly enhance bacterial delivery and colonization within diseased tissues [50]. Consequently, the development of programmable, multifunctional surface engineering strategies is essential to overcome current translational bottlenecks and fully realize the therapeutic potential of bacterial-based treatments.

## Bacterial surface modification

**Fig. 2:**
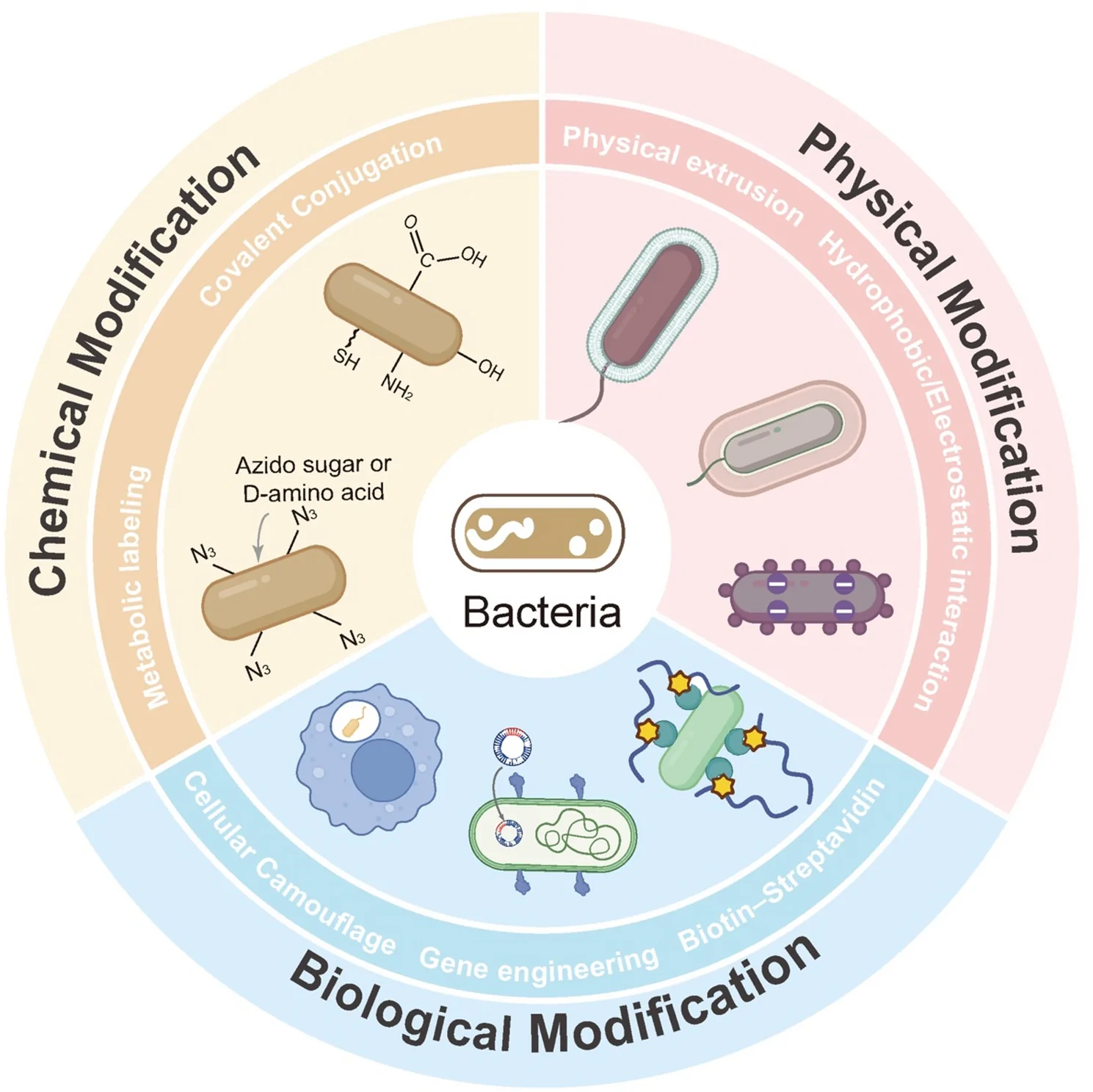
Schematic illustration of bacterial surface modification strategies. A comprehensive overview of physical, chemical, and biological approaches for engineering the bacterial surface. Physical methods are primarily achieved through membrane extrusion or by leveraging hydrophobic and electrostatic interactions. Chemical strategies encompass both covalent conjugation and metabolic labeling techniques. Biological modifications, meanwhile, include the use of cell-camouflaged bacteria, the biotin–streptavidin system, and genetic engineering

Bacterial surface modification refers to strategies that engineer the surface properties of bacteria to overcome biological barriers encountered during in vivo delivery, thereby enhancing effective delivery and improving their accessibility to diseased sites. Such approaches endow bacteria with novel and customizable functionalities beyond their native capabilities. Based on their underlying mechanisms, bacterial surface modification strategies can be categorized into three major types: physical modification, chemical modification, and biological modification **(**Fig. 2**)**. Physical modification typically exploits non-covalent interactions, such as membrane coating via mechanical compression or adsorption through electrostatic or hydrophobic forces, to confer new surface functionalities. Chemical modification establishes covalent bonds between exogenous molecules and bacterial surface groups, thereby ensuring stable immobilization of functional moieties. Biological modification leverages endogenous biological processes or genetic engineering, and can be further divided into two approaches: (i) exploiting natural biological interactions or camouflage mechanisms to achieve biological coupling on the bacterial surface, and (ii) programming bacteria at the genetic level to biosynthesize and display specific functional proteins or ligands on their outer membrane. In summary, physical modification emphasizes convenient physical adsorption or structural encapsulation; chemical modification focuses on stable covalent conjugation; whereas biological modification relies on intrinsic biological processes or genetic regulation to drive autonomous surface functionalization.

### Physical modification

#### Cell membrane encapsulation

Cell membrane encapsulation is a non-specific approach that uniformly modifies the entire bacterial surface. Typically implemented through extrusion or ultrasonication, this technique involves wrapping bacteria with exogenous membranes. Membrane fragments disrupted during extrusion subsequently reassemble around the bacteria. Natural cell membranes largely retain the intrinsic components and biological functions of the donor cells, enabling superior biocompatibility that protects bacteria from immune attack and harsh physiological conditions.

Erythrocyte membranes exemplify an ideal encapsulation material due to their inherent low immunogenicity, anti-phagocytic properties, and prolonged circulation. To fabricate cell membrane-coated bacteria, Cao et al. isolated erythrocyte membranes from murine blood, mixed them with EcN, and extruded the mixture 11 times through a 1-µm polycarbonate membrane using a mini-extruder [51]. The “self-marker” protein CD47 present on erythrocyte membranes [52, 53] further reduces macrophage-mediated clearance, significantly enhancing bacterial survival in the bloodstream. Notably, oval-shaped *Enterococcus faecalis* and spherical *Staphylococcus aureus* can also be readily encapsulated, highlighting the broad applicability of erythrocyte membrane coating across diverse bacterial morphologies. In addition to erythrocyte membranes, other natural membranes—such as yeast [54] and platelet membranes [55]—have been widely explored to endow bacteria with distinct functionalities. Macrophage membranes, enriched with surface receptors, have recently drawn substantial interest. Zhang et al. applied ultrasonication and co-extrusion to encapsulate BCG within membranes derived from RAW264.7 macrophages, producing a macrophage membrane–camouflaged BCG construct (M@BCG) that effectively evaded immune clearance while enhancing tumor targeting capabilities. Moreover, this membrane camouflage was shown to drive the M1 polarization of bone marrow-derived macrophages (BMDMs) and substantially enhance major histocompatibility complex II (MHC-II) expression compared to unmodified BCG [56]. However, it is important to note that the RAW264.7 source cells utilized in this study are typically considered to be in an unpolarized (M0-like) resting state. While these M0-like membranes successfully confer immune evasion properties and enhance tumor accumulation, they do not inherently possess the active immunomodulatory capabilities characteristic of polarized macrophages. This represents a notable limitation of the current design and a crucial avenue for future optimization. Given their high degree of plasticity, macrophages are profoundly shaped by varying pathological microenvironments, making phenotypic modulation a compelling therapeutic paradigm [57, 58]. Functionally, M1-type macrophages exert potent anti-tumorigenic effects by secreting pro-inflammatory cytokines (e.g., IL-1β, TNF-α) and chemokines (e.g., CXCL9, CXCL10) [59]. Conversely, M2-type macrophages foster an anti-inflammatory, pro-angiogenic, and tissue-repairing milieu; their secretion of immunosuppressive factors, such as IL-10 and TGF-β, impedes cytotoxic T lymphocyte activity, ultimately driving tumor progression and metastasis [60]. Consequently, employing membranes derived from M1-polarized macrophages to camouflage and deliver therapeutic nanomedicines [61]—thereby maximizing their capacity to re-educate tumor-associated macrophages (TAMs)—emerges as a highly promising and superior strategy. Despite these advantages, challenges such as limited membrane extraction efficiency and the potential shedding of surface membrane coatings during bacterial division, which may introduce uncontrollability, could constrain large-scale application.

#### Electrostatic/hydrophobic interactions

The inherent electronegativity and hydrophobicity of bacterial surfaces provide a versatile foundation for engineering modifications through non-covalent interactions. Electrostatic interactions are among the most widely utilized. Due to the abundance of phosphate groups and acidic residues (e.g., carboxyl and hydroxyl groups), bacterial surfaces are predominantly negatively charged [62]. Consequently, positively charged materials—including cationic polymers and nanoparticles—readily bind to live bacteria, conferring diverse exogenous functionalities [63]. In addition to macromolecules, positively charged metal ions can also serve as bridging agents to drive the self-assembly of anions on bacterial surfaces [64], or undergo biomineralization to form insoluble protective coatings in situ, thereby shielding bacteria from environmental stress [65]. Utilizing these strategies, researchers have developed a series of oral probiotic formulations for treating gastrointestinal disorders [66, 67]. However, the relatively weak nature of electrostatic interactions often leads to unstable or heterogeneous coatings. To overcome these limitations, Anselmo et al. developed a layer-by-layer encapsulation technique, enabling the sequential self-assembly of cationic chitosan and anionic alginate on *Bacillus coagulans*, thus providing robust gastrointestinal protection while preserving bacterial viability [68]. This strategy is broadly applicable to various charged polymers and probiotic strains. For Gram-negative bacteria, the outer membrane composed of a lipid bilayer serves as an anchor for hydrophobic interactions. The hydrophobic domains of amphiphilic polymers (e.g., DSPE) can insert into the outer membrane, forming a protective surface layer that delays immune clearance. Their hydrophilic termini can be functionalized with fluorescent dyes, targeting peptides, or bioorthogonal groups for subsequent modification [46, 69]. Although non-covalent strategies are simple and broadly compatible, their limited interaction strength often results in insufficient stability, restricting their applicability in advanced bacterial engineering.

### Chemical modification

#### Covalent conjugation

Covalent conjugation strategies exploit the abundant reactive groups present on bacterial surfaces to stably tether functional molecules through controlled chemical reactions. In Gram-negative bacteria, lipoproteins (LPPs) and β-barrel outer membrane proteins dominate the surface proteome, whereas Gram-positive bacteria display a broader diversity of surface proteins [70, 71]. These proteins contain multiple reactive groups—primary amines (lysine side chains and N-termini), carboxyl groups, thiols (cysteine residues), and hydroxyl groups—that can serve as chemical anchors. Common covalent modifications include aminoacylation, carboxyamidation, and thiol-based Michael addition, all of which proceed under mild conditions with high coupling efficiency [72].

Amino groups readily react with electrophilic reagents under physiological conditions. Common acylating reagents include N-hydroxysuccinimide (NHS) esters and trichloride-activated molecules [73]. NHS esters react with surface-exposed amines under mildly basic conditions to form stable amide bonds [74], enabling conjugation of nanomaterials [75], small-molecule drugs [76], and polymers [77]. They can also mediate functional group conversion, broadening the library of surface-modifiable molecules. For instance, after biotinylating bacterial surfaces using NHS-biotin, subsequent application of the biotin–streptavidin system facilitates rapid and versatile labeling with diverse functional molecules [78, 79].

Free carboxyl groups on surface proteins can be activated by1-ethyl-3-(3-dimethylaminopropyl) carbodiimide (EDC) and NHS, forming intermediates that react with amine-containing molecules to generate amide bonds [80]. Using this strategy, Geng et al. conjugated tumor-specific aptamers to the surface of VNP20009 through one-step amidation, enhancing tumor colonization [81]. Similarly, EDC/NHS-mediated reactions allow conjugation of carboxylated, drug-loaded liposomes to amine-rich magnetotactic bacteria for targeted delivery [82]. The thiol group in cysteine residues exhibits strong nucleophilicity and participates in Michael addition reactions with α,β-unsaturated carbonyls to anchor functional groups onto bacterial surfaces [83]. Maleimide reagents are widely used in such applications. For example, maleimide-functionalized upconverting nanoparticles (UCNPs) readily conjugate to thiol-containing engineered bacteria to enable synergistic photodynamic therapy [84]. Compared with amine- or carboxyl-based modifications, thiol-based reactions provide orthogonal functionalization pathways, facilitating multi-site or spatially controlled engineering. Luo et al. developed a cell-compatible iminoester reaction to convert abundant surface amines in EcN into free thiols, thus enabling spontaneous disulfide exchange with mucosal polydisulfides to enhance stable colonization in mucus-rich tissues [85].

Beyond direct reactions with native functional groups, dopamine-assisted deposition and enzyme-catalyzed conjugation offer additional covalent modification routes. Dopamine undergoes self-polymerization under mild conditions to form a polydopamine (PDA) coating that adheres to bacterial surfaces via covalent interactions [86]. This PDA layer enables further conjugation of functional molecules through Michael addition or Schiff-base chemistry. Dopamine can also co-deposit with biomolecules in one-pot reactions, preserving bioactivity while simplifying the modification process [87, 88]. Alternatively, exploiting the strong adhesive properties of PDA, a biohybrid system has been developed that anchors nanoparticles bound to the surface of anaerobic Bifidobacterium, enabling the targeted delivery of therapeutic nanomedicines to the hypoxic regions of solid tumors [89]. Enzymatic strategies provide higher selectivity and efficiency compared with spontaneous chemical reactions. Jia et al. reported a tyrosinase-catalyzed oxidative coupling method to conjugate phenol-labeled probes to teichoic acids on Gram-positive bacteria [90]. In addition, glycosyltransferases and peptidyl transferases can also mediate enzymatic surface labeling. Generally, covalent surface modification offers high stability; however, certain covalent conjugation strategies may compromise bacterial viability due to the introduction of bulky chemical moieties or the use of harsh reaction conditions.

Therefore, it is essential to select covalent modification approaches that minimize adverse effects on bacterial activity for practical applications. To mitigate viability loss, optimizing reaction parameters—such as avoiding organic solvents, maintaining near-neutral pH, shortening incubation times, and minimizing the steric hindrance of introduced chemical moieties—is highly beneficial. Furthermore, the integration of mild and stable covalent modification strategies, notably the SpyTag/SpyCatcher system, has revolutionized bacterial surface functionalization. Derived from the CnaB2 domain of the fibronectin-binding protein FbaB in Streptococcus pyogenes [91, 92], this system splits the domain into a 13-amino-acid peptide (SpyTag) and a 116-amino-acid protein partner (SpyCatcher) [93]. Upon mixing, the two components spontaneously reconstitute to form an irreversible isopeptide bond across a remarkably broad range of conditions, spanning 4–37 °C, pH 5–8, diverse buffers (without specific anion or cation requirements), and even non-ionic detergents [94]. This approach yields a linkage highly resilient to extreme pH, elevated temperatures, and proteolytic degradation [95]. Crucially, its modular versatility enables a highly efficient “two-step assembly” strategy, which is particularly advantageous for conjugating bulky macromolecules or nanotherapeutics. By initially displaying the ultra-short SpyTag on the bacterial surface, the system introduces negligible steric hindrance, thereby fully preserving normal bacterial proliferation and membrane integrity. Subsequently, massive functional modules fused with SpyCatcher can be seamlessly anchored onto the living bacteria through a simple co-incubation step. This approach effectively circumvents the severe membrane stress and viability loss typically associated with direct physical coating or massive chemical conjugations, significantly streamlining the construction of robust, multifunctional bacterial delivery systems.

#### Metabolic labeling

Metabolic labeling is a strategy that leverages intrinsic biosynthetic pathways to incorporate exogenous small-molecule monomer conjugates into cell membrane polymers, thereby enabling bacterial surface modification [96]. This approach typically includes one-step or two-step labeling. Compared with one-step labeling—where functional molecules are directly integrated—two-step labeling first introduces an exogenous monomer containing a bioorthogonal group into the target polymer, followed by conjugation with complementary functional molecules. Due to their smaller size and reduced steric hindrance, the bioorthogonal groups used in two-step labeling generally exhibit higher incorporation efficiency and allow the installation of larger functional moieties. Bioorthogonal click chemistry is one of the most widely applied linkage strategies in metabolic tagging and has been extensively used across multiple bacterial species, including *Escherichia coli*, *Salmonella Typhimurium*, and *Clostridium butyricum* [97–99].

Surface polysaccharides represent major targets for metabolic labeling. Metabolic glycoengineering introduces non-native sugars into cell membranes, enabling efficient site-specific conjugation of functional molecules through chemical reactions such as azide-dibenzocyclooctyne (DBCO) click chemistry [100–102]. Bacterial surfaces contain diverse polysaccharides—including capsular polysaccharides, peptidoglycans (PG), glycolipids, and glycoproteins—providing ample substrates for metabolic incorporation [96]. PG is a ubiquitous component of bacterial cell walls, consisting of polysaccharide chains crosslinked by stem peptides enriched in D-amino acids [103]. The complexity of PG biosynthesis allows selective incorporation of D-amino acid derivatives into bacterial surfaces [104] without affecting host cells. Functional molecules can then be displayed through one- or two-step metabolic labeling. For example, Ji et al. incorporated azide-modified D-alanine (D-Ala) into the cell wall of *Lactococcus lactis* and subsequently attached dopamine-β-glucan conjugates via click chemistry, successfully constructing functionalized engineered probiotics [105].

Beyond PG, components such as lipopolysaccharide (LPS) and glycoproteins can serve as metabolic labeling anchors. Studies have shown that the endogenous L-fucose synthesis pathway in the O-antigen of LPS can be replaced by a heterologous rescue pathway, enabling incorporation of azide-functionalized L-fucose, followed by two-step metabolic labeling to achieve luciferin surface modification [106]. Similarly, metabolic labeling by substituting the LPS core sugar 3-deoxy-D-manno-oct-2-ulosonic acid (Kdo) with an azide-bearing analogue provides a chemical handle for conjugating fluorescent dyes via click chemistry, enabling specific bacterial imaging [107]. In contrast to sugar metabolism-based strategies, protein-based metabolic labeling uses genetic code expansion (GCE) to incorporate noncanonical amino acids (ncAAs) with bioorthogonal groups into surface-exposed proteins. Subsequent bioorthogonal chemical reactions enable efficient and site-specific surface functionalization [108]. For instance, expression of orthogonal tRNAᵗʸʳ–tyrosyl-tRNA synthetase (TyrRS) pairs expressed in methionine-deficient *Escherichia coli* allows site-specific incorporation of ketone-bearing ncAAs in response to amber codons, followed by in vivo labeling through reaction with hydrazine-functionalized fluorophores [109]. Overall, metabolic labeling offers advantages such as biological orthogonality, mild reaction conditions, operational simplicity, and negligible impact on bacterial viability, and has therefore emerged as one of the most widely adopted strategies for chemical surface modification.

### Biological modification

#### Cell-camouflaged bacteria

Conventional intravenous bacterial therapy relies on the passively leaky tumour vasculature for initial tumour accumulation [10], a process which presents a bottleneck for efficient and specific delivery. To overcome this, an active targeting approach has been devised by loading bacteria into live macrophages. This cellular “camouflage” serves a dual purpose: it conceals the bacteria to evade premature clearance and actively co-opts the macrophages’ intrinsic tumour-homing ability for precise delivery, thereby enhancing bacterial tumor accessibility. To this end, mouse peritoneal macrophages or RAW264.7 cells were co-incubated with VNP20009. This one-hour phagocytosis process successfully generated a cellularly camouflaged bacterial system with an ideal payload, while maintaining high host cell viability [110]. This approach markedly reduced nonspecific bacterial dissemination to normal tissues. To improve the translational potential, the same group established a macrophage carrier system based on liquid nitrogen cryopreservation. Cryopreservation eliminates microbial pathogenicity while retaining macrophage function, addressing challenges associated with cell sourcing and enabling scalable production of safe, clinically compatible carrier cells. This strategy provides a highly promising platform for targeted bacterial delivery [111]. To further enhance the precision and controllability of this therapeutic strategy, the researchers engineered a controllable autolysis system in macrophages, enabling regulated pyroptosis upon reaching the tumor site. This allows for rapid and substantial release of intracellular bacterial cargo and pro-inflammatory cytokines, thereby improving both the efficacy and safety of the treatment [112].

Cell-based therapies have recently achieved major breakthroughs in oncology, with CAR T therapy attracting global interest. CAR T cells are genetically engineered to precisely recognize and eliminate tumor cells, and several CAR T products have been approved worldwide, demonstrating strong clinical efficacy in hematologic malignancies [113]. However, major obstacles remain for solid tumor treatment, including immunosuppressive tumor microenvironments, antigen heterogeneity, and limited intratumoral infiltration. In contrast, macrophage-based engineered therapies hold unique advantages. Owing to their intrinsic ability to infiltrate solid tumors, macrophages may more effectively traverse tumor stromal barriers [114]. This has inspired efforts to genetically engineer macrophages to improve their tumor tropism and therapeutic potency. One such approach involves engineering macrophages with a synthetic receptor inspired by CAR design. In this construct, macrophages are programmed to express the arginine-glycine-aspartate (RGD) peptide on the surface, which binds to the tumor-associated alpha v beta 3 (αvβ3) integrin. This binding facilitates the active migration and homing of the engineered macrophages to tumor sites. Thus, by utilizing these engineered macrophages as living vectors, bacteria are not only delivered with enhanced tumor tropism but also protected during transit. Bacteria phagocytosed by these engineered macrophages are released following carrier-cell lysis within the tumor microenvironment, subsequently exerting therapeutic effects such as oncolysis or immune activation [11]. This temporary phagocytic camouflage broadens the conceptual space for bacterial surface engineering. This modification strategy offers operational simplicity, requiring only a straightforward co-incubation step to achieve surface engineering without compromising bacterial viability. Notably, unlike other approaches that rely solely on genetic or chemical engineering of the bacteria themselves, this method harnesses the intrinsic tumor-homing and infiltration capabilities of macrophages, thereby enhancing the active tumor-targeting properties of the bacteria. Additionally, the macrophage-mediated camouflage reduces bacterial clearance in the bloodstream, further improving systemic delivery efficiency. Another innovative approach constructs a non-endocytic bacterial complex that adheres stably to the cell surface. An et al. developed a polysaccharide-mediated bioadhesion method to anchor EcN onto the external surface of RAW264.7 macrophages, forming a “cell backpack” structure [115]. In this configuration, the bacteria continuously activate the macrophage carrier through pathogen-associated molecular patterns, promoting polarization toward an anti-tumor phenotype. Simultaneously, bacteria delivered to the tumor microenvironment proliferate locally, sustaining strong anti-tumor immune responses and achieving synergistic therapeutic outcomes.

#### The biotin-streptavidin system

Owing to its extraordinary affinity and robust stability, the biotin–streptavidin system has become a cornerstone for functionalizing bacterial surfaces. This platform enables the precise, modular conjugation of therapeutic cargoes, from small molecules to biomacromolecules, onto bacteria [116]. Unlike encapsulating strategies that can impair native cellular functions, this approach minimally interferes with the bacterium’s vital activities, such as chemotaxis and environmental interaction, which are essential for targeted therapy [117]. The durability of the biotin-streptavidin interaction under diverse conditions further facilitates the construction of sophisticated, multi-functional bacterial hybrids. A typical strategy involves anchoring biotin onto bacterial outer membrane proteins through bioconjugation while linking streptavidin to therapeutic payloads such as drugs or nanoparticles. This step enables the modular surface modification of bacteria through specific biotin–streptavidin binding.

This modular strategy enables highly adaptable bacterial delivery systems. For example, recent work loaded paclitaxel into streptavidin-modified liposomes while displaying biotinylated outer membrane proteins on attenuated *Salmonella*, forming an actively targeted bacteria-driven drug delivery system that significantly enhanced intratumoral drug accumulation [118]. Similarly, Suh et al. conjugated streptavidin to polylactic-polyhydroxybutyric acid copolymer nanoparticles and modified the surface of *Salmonella* VNP20009 with biotinylated polyclonal antibodies. The specific interaction enabled efficient nanoparticle loading, producing a multifunctional platform integrating tumor targeting, therapy, and imaging [119]. This strategy has also been applied to BCG surface engineering. Researchers prepared streptavidin-modified, drug-loaded nanoparticles and simultaneously functionalized BCG with a biotinylated *Mycobacterium tuberculosis* antibody (B-Anti-MT). Through biotin–streptavidin bridging, a drug-loaded BCG complex was created, achieving synergistic effects between the vaccine carrier and chemotherapeutic agents [120].

#### Genetic engineering of bacterial outer membranes

Genetic engineering can stably anchor functional molecules—including exogenous proteins, peptides, and enzymes—onto the bacterial outer membrane through surface-display systems, thus expanding the functional repertoire of engineered bacteria. This typically involves inserting the target sequence into a specific domain of an anchor protein or fusing it to the N- or C-terminus of the anchor protein, enabling surface localization through endogenous protein transport pathways.

Bacterial Outer membrane proteins possess conserved β-barrel structures composed of antiparallel β-strands forming short periplasmic loops and longer surface-exposed loops. These accessible extracellular loops provide ideal sites for displaying exogenous functional motifs. Leveraging the exposed loops of outer membrane proteins as display scaffolds, Wu et al. engineered *Salmonella* to express a fusion of OmpA with the SpyTag peptide on its surface. SpyTag then forms a spontaneous, covalent bond with its partner protein SpyCatcher. To optimize this system for targeting, the researchers used a truncated SpyCatcherΔ fused to an RGD peptide. This design enabled dose- and time-dependent functionalization, Incubation of 3 mg of SpyCatcherΔ with 10^⁸^ CFU bacteria for one hour achieved uniform, saturated surface display of the engineered protein [121]. The SpyTag/SpyCatcher system offers several advantages, including rapid spontaneous isopeptide bond formation under physiological conditions without additional catalysts, stable covalent surface display that minimizes detachment during in vivo delivery, and modular “plug-and-play” functionalization flexibility. This system offers distinct advantages for the display of small- to medium-sized proteins. Similarly, Bi et al. engineered an outer membrane modification system using OmpA fused with the Asn‑His‑Val (NHV) tripeptide motif recognized by the plant-derived peptide ligase Butelase1. Expression of this construct in *E.coli* BL21 enabled stable display of the NHV motif. Butelase1 then catalyzed highly efficient covalent ligation of diverse functional molecules—including fluorophores, biotin, and glycosylated tumor-associated peptides—onto the bacterial surface [122]. Moreover, bacterial ice nucleation proteins (INPs) serve as another widely employed scaffold for protein surface display. For example, fusion of an INP tag to the histone-like protein HlpA enabled its presentation on the surface of EcN, significantly enhancing bacterial adhesion to heparan sulfate proteoglycans on colorectal cancer cells [123].

A more direct strategy involves the precise genetic regulation of endogenous bacterial surface components. While simple knockout approaches—such as deleting flagellar genes—can reduce immunogenicity, they often compromise tumor targeting and colonization abilities, making them suboptimal. Consequently, research has shifted toward spatiotemporally controlled modulation of specific components, such as immunogenic extracellular polysaccharides (EPS). Notably, deletion of *htrA* significantly reduces EPS levels in VNP20009 without impairing tumor colonization. Based on this observation, researchers designed a quorum-sensing regulatory circuit enabling engineered bacteria to maintain low EPS expression in normal tissues—facilitating clearance—while restoring EPS production within tumors to locally potentiate antitumor immune responses [24].

## Therapeutic disease types

To date, a diverse range of surface-engineered bacteria has been developed. Compared with their unmodified counterparts, these engineered strains can integrate multiple exogenous functions and have been applied in treating various disease types **(**Table 4**)**.

**Table 4:** Application of surface modified bacteria in treating diseases

Disease model	Induction method	Bacterial strain	Modification strategy	Modification material	Intended purpose	Therapeutic outcome	Reference
Inflammatory bowel disease	Dss or salmonella typhimurium infection	*E.coli* Nissle 1917 (EcN)	Supramolecular self-assembly	Lipid membrane	Improve survival	Alleviated colon shortening, inflammation, and body-weight loss	[67]
	K88 and salmonella infection	*E.coli* Nissle 1917 (EcN)	Ionic binding	Enteric polymer : Eudragit l100-55	Improve targeting	Increased beneficial bacteria and reduced pathogen colonization	[173]
	5-fu–induced jejunal mucositis	*E.coli* Nissle 1917 (EcN)	Surface thiolation	Free thiols	Enhance colonization	~ 170-fold higher adhesion in mucin-rich jejunum	[174]
	Dss induction	*E.coli* Nissle 1917 (EcN)	Electrostatic/hydrogen bonding	Oxidized starch (os), polyethylenimine (pei)	Enhance colonization	Significant therapeutic effect, minimized weight loss, reduced colon shortening and dai, enhanced oral delivery efficiency	[175]
	Dss induction	*B. Longum* BF839	Electrostatic interaction	Caco₃ coating	Oral probiotic therapy	Increased colon length, reduced tnf-α levels	[65]
	Dss induction	*E.coli* Nissle 1917 (EcN)	Electrostatic interaction	Fe(no₃)₃·9 h₂o, chitosan, fucoidan	Improve targeting; reduce inflammation	Suppressed neutrophil activity and pro-inflammatory macrophage polarization	[176]
Antibiotic-associated diarrhea(aad)	Levofloxacin induction	*E.coli* Nissle 1917 (EcN)	Electrostatic interaction	Tannic acid, fe³⁺	Enhance antibiotic resistance	Lower il-6, il-1β, tnf-α; increased il-10	[66]
Diabetes	High-fat diet	*E.coli* Nissle 1917 (EcN)	Covalent conjugation	Polyethylene glycol	Improve safety	Improved glucose homeostasis, glucose tolerance, and insulin resistance; reduced adipose inflammation	[145]
Subcutaneous solid tumors	CT26, B16F10	*E.coli* Nissle 1917 (EcN)	Covalent conjugation	Αpd1 antibody and sars-cov-2 spike protein are used as immune checkpoint inhibitors and virus-specific antigens	Regulate immune responses	Ecn-αpd1-s1 induced a strong humoral and cellular immune response, and the tumor regression was significantly enhanced.	[177]
	MET6	*E.coli* Nissle 1917 (EcN)	Bioorthogonal chemistry	Dbco-modified sialidase	Catalytic activity	Enhanced t-cell/nk infiltration and m1 macrophage repolarization; suppressed tumor growth	[178]
	4T1、H22	*E.coli* Nissle 1917 (EcN)、*S. typhimurium* (VNP20009)	Amide covalent coupling	Aptamers	Improve targeting	2–4× accumulation in tumors; activated antitumor immunity; inhibited tumor growth	[81]
	CT26	*S. oneidensis* MR-1	Mineralization, covalent modification	Pd nanoparticles + mb-loaded zif-90	Photothermal therapy	Enhanced tumor targeting and photothermal ablation	[179]
	CT26	*E.coli* Nissle 1917 (EcN)	Click chemistry	Magnetic nanoparticles	Magnetothermal ablation	Combined magnetothermal therapy improved tumor suppression	[180]
	4T1	*E.Coli* BL21	Surface thiolation, click chemistry、covalent conjugation	Ucnp nanoconverters	Combined photothermal therapy	Induced systemic immune response and tumor apoptosis; prolonged survival	[181, 182]
	MB49	*Salmonella* Yb1	Amide conjugation	Icg-loaded nanophotosensitizers (inps)	Combined photothermal therapy	Low-dose and high-efficiency photothermal therapy is realized, the therapeutic dose is reduced to about 1/5 of the reported inps, and the tumor inhibition rate is 100%.	[131]
	CT26	*E.coli* Nissle 1917 (EcN)	Electrostatic interaction	Hsa-based nano-drug carrying paclitaxel (ptx) and bay-876	Metabolic combination therapy	Disrupted tumor glucose availability, enhanced targeting/uptake, inhibited tumor growth	[183]
Breast cancer	MDA-MB-231	*B. Longum*	Click chemistry	Plga nps with low boiling point perfluorohexane (pfh) liquid as the core.	Ultrasound-assisted drug delivery	. Improved energy deposition, enhanced imaging and therapeutic efficacy	[130]
Orthotopic breast cancer	4T1	*S. typhimurium* (VNP20009)	Biotin-avidin conjugation	Nanoparticles	Drug loading	~ 100-fold enhanced tumor retention and distribution	[129]

*S. typhimurium: Salmonella typhimurium;; E. coli: Escherichia coli; BCG: Bacillus Calmette-Guérin; B. Longum: Bifidobacterium longum*; *S. oneidensis: Shewanella oneidensis*

### Surface-engineered bacteria for tumor therapy

#### Enhancing the safety of bacterial therapy

Successful clinical translation of bacterial therapeutics requires preserving their antitumor efficacy while stringently minimizing the risks of systemic infection and excessive immune activation. In this regard, surface engineering aimed at improving biosafety has become a central research priority. Encapsulating bacteria with complete, biocompatible coatings represents an effective strategy. Such coatings not only shield bacteria from premature immune recognition and rapid clearance but also, depending on the material or method used, endow passive or active tumor-targeting properties.

**Fig. 3:**
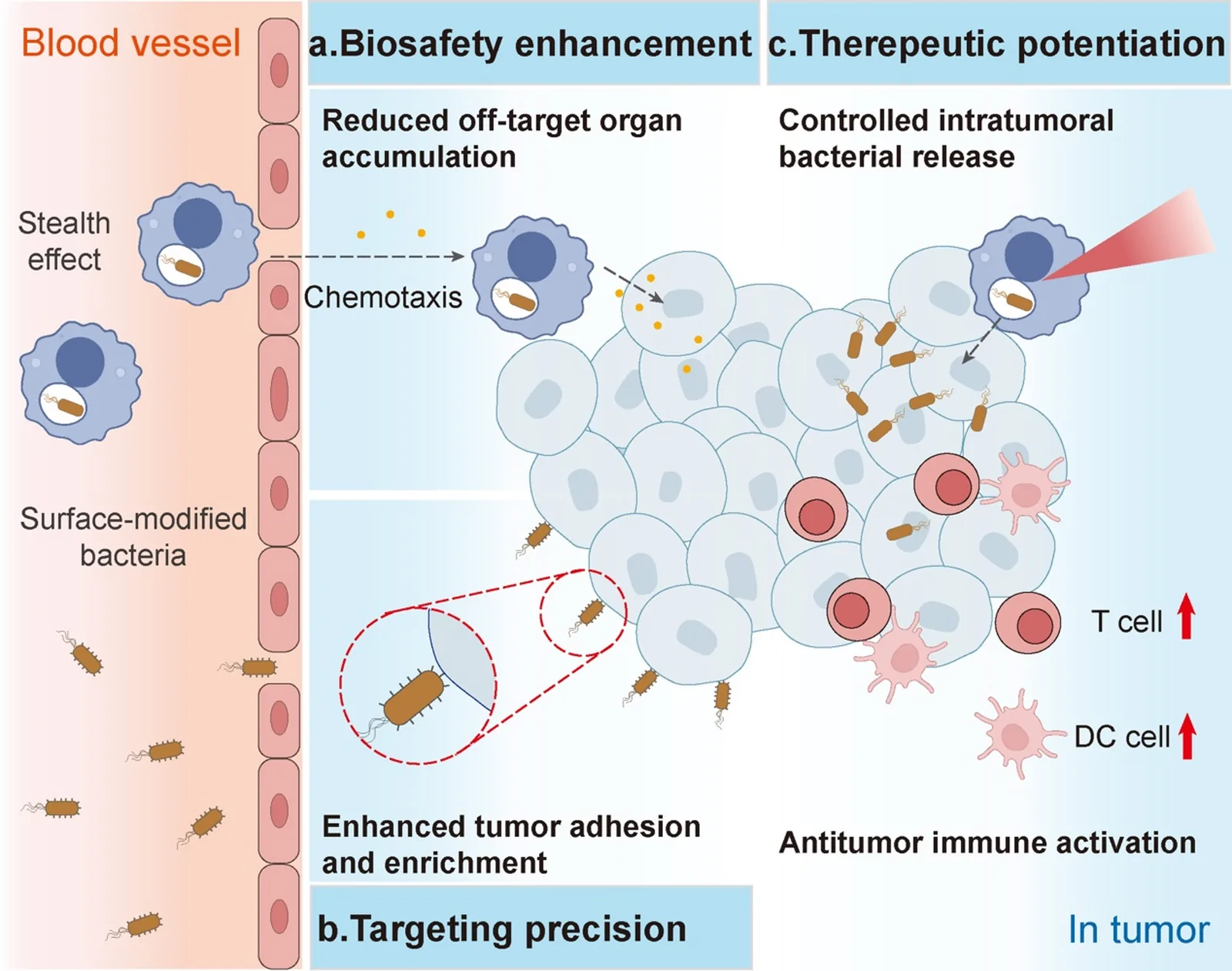
Schematic overview of surface-engineered bacteria for enhanced tumor therapy**.** This illustration depicts the multifaceted design and therapeutic mechanisms of surface-engineered bacteria following intravenous administration, navigating from the bloodstream into the tumor microenvironment. (**a**) Enhancing biosafety via surface engineering. In contrast to unmodified bacteria, cell-camouflaged bacteria, such as those encapsulated within macrophages, effectively evade immune surveillance in the bloodstream. These macrophage–bacteria hybrids actively migrate along chemokine gradients toward the tumor, thereby reducing non-specific accumulation and off-target toxicity in healthy organs. (**b**) Augmenting targeting precision via surface engineering. Functionalizing the bacterial surface with tumor-specific ligands, RGD peptides or adhesins promotes selective adhesion to tumor cells and enhances targeted enrichment at the tumor site. (**c**) Boosting therapeutic efficacy through controlled release and immune activation within the tumor. Advanced surface modification techniques enable precise spatiotemporal control over bacterial distribution, leading to rapid and controllable intratumoral release and proliferation. This localized bacterial expansion potently activates antitumor immune responses, thereby driving durable therapeutic efficacy

A variety of biomaterials—including lipid layers [124], biopolymers [125], and natural cell membranes (e.g., erythrocyte [51], yeast [54], platelet [55], and macrophage membranes [56])—have been successfully employed as coating materials. By masking pathogen-associated molecular patterns (PAMPs), these coatings facilitate immune evasion, thereby reducing the risk of cytokine storms and acute toxicities (such as hepatic and splenic injury) associated with systemic bacterial delivery. Studies have shown that appropriately coated bacteria, when administered intravenously, display limited retention and replication in healthy tissues, yet exhibit enhanced tumor homing and colonization, thereby improving the therapeutic window. For example, Wu et al. devised a macrophage-camouflaging delivery strategy based on the intrinsic ability of Salmonella to survive within macrophages by forming Salmonella-containing vacuoles (SCVs). Instead of passive encapsulation, this approach internalizes bacteria into macrophages, effectively concealing PAMPs and enabling evasion of innate immune surveillance. This immunological camouflage markedly decreases bacterial clearance and attenuates acute systemic inflammatory toxicity, mitigating liver and spleen damage as well as treatment-associated weight loss **(**Fig. 3a**)** [11]. In another study, Geng et al. conjugated tumor-specific aptamers onto bacterial surfaces using a simple, cell-compatible amidation reaction, substantially enhancing tumor colonization following systemic administration. Notably, inflammatory responses at 60 h post-injection remained comparable to the PBS control, indicating the favorable biosafety profile of surface-modified bacteria in tumor therapy [126].

#### Augmenting the targeting precision and accumulation efficiencyof bacterial therapy

The therapeutic efficacy of bacterial therapies critically depends on adequate bacterial accumulation at disease sites. However, most bacterial strains lack sufficient intrinsic targeting capacity, resulting in limited tumor enrichment. Thus, improving bacterial targeting and aggregation at pathological sites is essential for optimizing therapeutic outcomes.

One approach involves engineering tumor-associated antigen–specific ligands on the bacterial surface to enhance adhesion to tumor cells. Genetic strategies can stably integrate single targeting peptides. For instance, PARK et al. engineered an attenuated, *ppGpp*-deficient *S. typhimurium* strain (*ΔppGpp*) to display RGD peptides on its surface. By specifically targeting the overexpressed αvβ3 integrin on tumor cells, this RGD-modified bacterium achieved enhanced tumor targeting and antitumor efficacy in mouse models of breast cancer (MDA-MB-231) and melanoma (MDA-MB-435) [127]. Another strategy employs modular surface-display systems to construct multivalent targeting peptides. Wu et al. established a multivalent RGD-targeting platform using attenuated Salmonella by first expressing an OmpA-SpyTag fusion protein as a bioconjugation scaffold. Through SpyCatcher/SpyTag covalent ligation, they efficiently displayed a targeting peptide containing four tandem RGD repeats (SC-RGD×4), generating the engineered strain AISI-ST/SC-RGD×4. The multivalent configuration significantly enhanced bacterial adhesion and tumor enrichment **(**Fig. 3b**)** [121]. Beyond peptides, adhesins represent another class of targeting molecules. A study from the Spanish National Center for Microbiology engineered synthetic adhesins onto the surface of *Escherichia coli* and assessed their affinity for cancer-associated antigens and tumor colonization in vivo. Compared with wild-type strains, adhesin-expressing *Escherichia coli* achieved robust colonization of solid tumors at lower doses while reducing nonspecific interactions with healthy tissues, thereby enabling precise tumor localization [128].

#### Boosting the therapeutic efficacy of bacterial agents

Improved tumor targeting inherently enhances therapeutic outcomes by increasing bacterial accumulation and, hence, drug release and immune stimulation at disease sites. A more direct approach, however, is to functionalize bacterial surfaces with therapeutic agents to confer immediate therapeutic activity.

##### Conjugation of small-molecule drugs

Small-molecule drugs can be immobilized on bacterial surfaces through physical adsorption or chemical conjugation, yielding efficient targeted delivery systems. These hybrids leverage the intrinsic tumor-tropism of bacteria to achieve highly localized drug enrichment, reduce systemic toxicity, and synergistically enhance bacteria-induced antitumor immune responses.

For example, via biotin–streptavidin binding, drug-loaded poly(lactic-co-glycolic acid) (PLGA) nanoparticles can be stably conjugated to *Salmonella*, resulting in an approximately 100-fold increase in nanoparticle retention and distribution within solid tumors [129]. Similarly, PLGA nanoparticles encapsulating low-boiling-point perfluorohexane (PFH) can be covalently linked to bacteria through carbodiimide chemistry. Upon exposure to high-intensity focused ultrasound (HIFU), these conjugates modulate the local acoustic microenvironment, enhancing energy deposition and improving therapeutic efficacy [130]. In another example, functionalized inorganic nanoparticles with carboxyl groups (–COOH) were covalently attached to the tumor-targeting, attenuated *Salmonella* strain YB1, generating YB1-INPs, a nanophotosensitizer–bacteria hybrid for photothermal therapy (PTT). This platform compensates for the limited intrinsic antitumor potency of natural bacteria and reduces the required dose of photothermal agents, enabling more precise, safer, and more efficient tumor ablation [131].

##### Conjugation of macromolecular therapeutics

Macromolecular agents such as antibodies, cytokines, and therapeutic enzymes exhibit potent biological activity but often face challenges including poor stability, limited tissue penetration, and high production costs. Leveraging bacteria as living delivery vehicles enables site-specific and sustained release of protein therapeutics within diseased tissues, thereby enhancing efficacy while reducing off-target effects. Although genetic engineering allows the surface display of simple peptides, anchoring complex functional proteins requires efficient bioconjugation platforms, such as the SpyCatcher/SpyTag system, to precisely immobilize macromolecules on the bacterial outer membrane. This strategy transforms engineered bacteria into “whole-cell biocatalysts” in vivo, enabling localized, high-efficiency catalytic therapy [132, 133].

##### Enhancing controllable bacterial release

Achieving precise, controllable bacterial release within lesions is essential for maximizing the therapeutic index and minimizing adverse effects. Beyond environmentally responsive coatings, externally triggered remote control represents a highly promising approach. Magnetic fields, ultrasound, microwaves, and chemical cues have been explored to program bacterial spatiotemporal behavior and enhance therapeutic performance.

Photothermal manipulation offers a direct means to modulate bacterial activity. However, conventional “bacteria-photothermal” coupling suffers from a key limitation: photothermal heating indiscriminately damages exposed bacteria, leading to substantial loss of viability, premature clearance, and inadequate deep-tumor colonization, ultimately impairing therapeutic durability. To address this challenge, Li et al. introduced a dual-gated macrophage-bacteria activation platform. The central concept is shifting the photothermal control switch from bacteria to their macrophage carriers. Under near-infrared irradiation, bacteria remain unharmed; instead, precise induction of immunogenic pyroptosis in macrophages triggers on-demand release of viable bacteria while simultaneously amplifying immune activation through pyroptotic danger signals [134]. In this way, the inherent bacteria‑induced macrophage pyroptosis is not merely preserved but strategically amplified, transforming a natural antitumor mechanism into an engineered, externally controllable event [135]. This design redefines the photothermal cue, from a nonspecific “kill signal” to a targeted “release-and-activate signal”, thereby elevating the modality from simple physical integration to biologically synergistic amplification and markedly improving the therapeutic index **(**Fig. 3c**)**.

### Surface-engineered bacteria for inflammatory bowel disease intervention

#### Improving bacterial survival and colonization/adhesion in the gut

The challenge of lesion accessibility is not unique to tumors. In inflammatory bowel disease (IBD), orally administered bacteria face a similarly harsh journey to reach the diseased tissue [136, 137]. Here, we discuss how surface-engineered bacteria address this accessibility issue in IBD intervention. Oral administration is the most common route for bacterial therapeutics targeting intestinal diseases. However, the harsh and dynamic gastrointestinal environment, including gastric acidity, pepsin, bile salts, digestive enzymes, gastric emptying, and intestinal peristalsis, severely reduces bacterial bioavailability and therapeutic effectiveness. Complete surface coatings have thus emerged as an effective strategy to enhance probiotic survival, colonization, and adhesion in the gut.

**Fig. 4:**
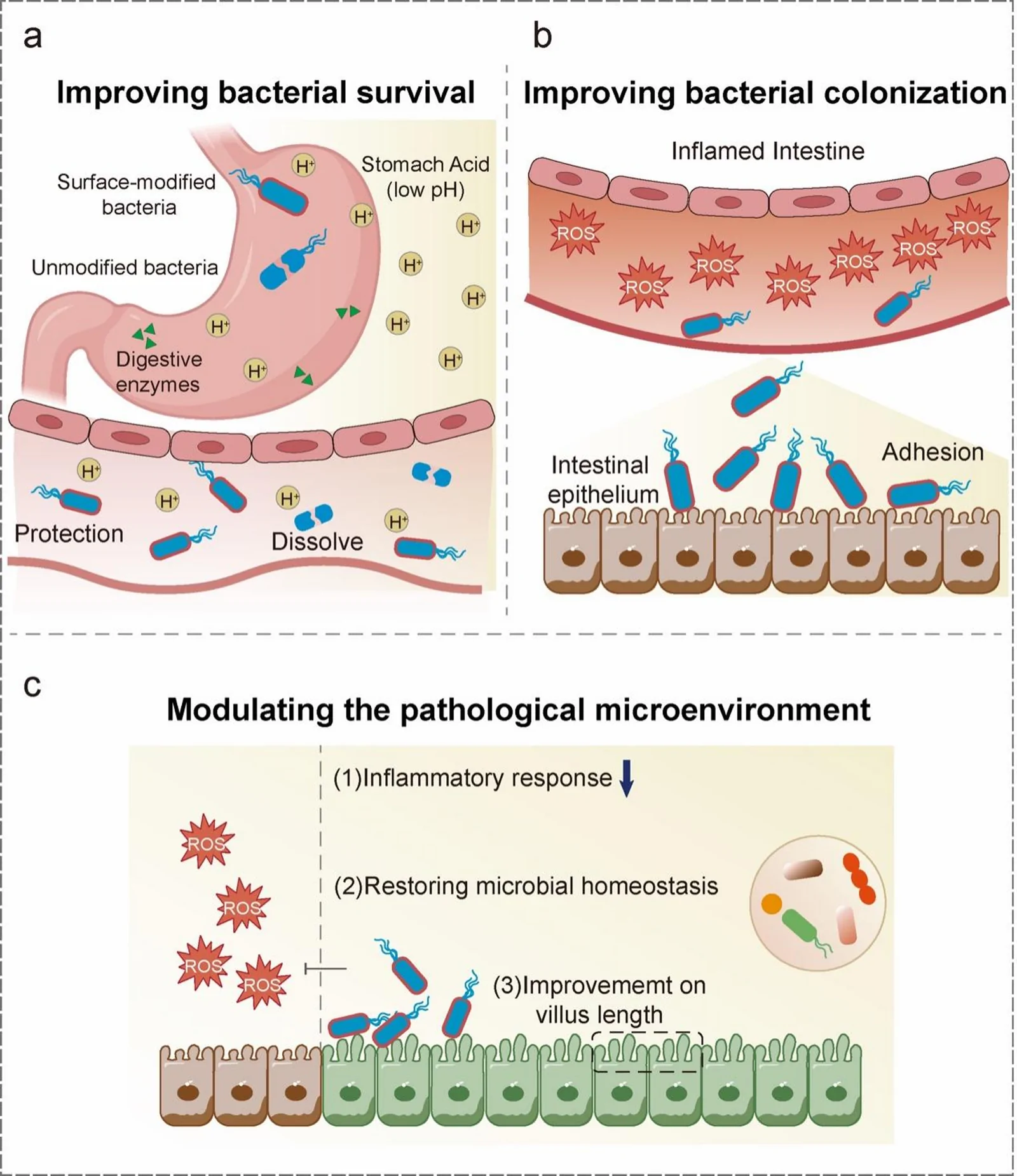
Surface-engineered bacteria for inflammatory bowel disease therapy.Schematic illustration of surface modification strategies to improve the therapeutic efficacy of orally administered probiotics in inflammatory bowel disease (IBD). (**a**) Surface engineering enhances bacterial survival in the gastrointestinal tract. Complete surface coatings protect probiotics from gastric acid and digestive enzymes, significantly improving intestinal survival. (**b**) Surface engineering augments bacterial colonization in the gut. Conjugation of synthetic adhesins onto bacterial surfaces promotes mucosal adhesion and alters gastrointestinal transit kinetics, increasing colonization and overall bacterial abundance. (**c**) Surface engineering modulates the pathological microenvironment. Decoration with ROS-scavenging materials enables simultaneous reactive oxygen species elimination, and microbiota regulation. This multifunctional approach effectively alleviates oxidative stress, reduces inflammatory cytokine levels, restores mucosal barrier integrity, and reestablishes microbial homeostasis in IBD models

Silk fibroin, a natural protein derived from silkworm cocoons, can transition from a random-coil to a β-sheet conformation, forming hydrogen bonds and hydrophobic interactions that assemble into robust nanoshells on diverse nanoparticle surfaces [138]. Leveraging this property, Hou et al. constructed complete silk-fibroin nanoshells on bacterial surfaces for mucositis treatment. Following oral administration, coated bacteria exhibited a nearly 52-fold improvement in intestinal survival and a 5.8-fold increase in colonization relative to unmodified bacteria [139]. Enhanced survival synergized with the intrinsic anti-inflammatory activity of silk fibroin, significantly augmenting therapeutic efficacy in mucosal inflammation **(**Fig. 4a**)**. Inspired by the biological role of adhesins in promoting colonization, Vargason et al. developed a modular platform in which bacteria were first biotinylated and subsequently conjugated to a synthetic adhesin (SA) via streptavidin coupling. This strategy was successfully applied to multiple strains—including *Lactobacillus casei*, *Escherichia coli*, *Bacillus coagulans*, and the commercial probiotic mixture Visbiome. SA-functionalized bacteria showed altered intestinal transit kinetics, enhanced colonization dynamics, and improved pharmacokinetics, enabling rapid establishment of local microenvironments, increased peak bacterial concentrations, and a 20% elevation in overall bacterial abundance**(**Fig. 4b**)**. [140].

#### Modulating the pathological microenvironment

Gastrointestinal diseases are frequently characterized by excessive oxidative stress and dysbiosis. Thus, scavenging reactive oxygen species (ROS) and restoring microbial homeostasis are critical to repairing mucosal injury and reestablishing intestinal equilibrium. Surface-engineering strategies have recently shown strong potential in enhancing the multifaceted therapeutic functions of probiotics in intestinal disorders.

To specifically address oxidative stress in IBD, Liu et al. designed a multilayer-engineered probiotic system. They first encapsulated EcN with a polydopamine coating to improve oral viability and mucosal adhesion, then conjugated ROS-scavenging hyaluronic acid-poly (propylene sulfide) nanoparticles onto the coated bacteria. The engineered system efficiently targeted inflamed sites and exerted a synergistic “physical protection-ROS elimination–microbiota modulation” trifunctional effect. This strategy alleviated oxidative injury while restoring microbial balance, achieving precise and effective treatment of IBD [141]. Similarly, Cao et al. reported an engineered *Bifidobacterium longum* strain (BL@B-SA50) created by anchoring single-atom iron catalysts (Fe SAs) onto the bacterial surface via C18-PEG-B bridges. Hydrophobic interactions between the C18 chain and Fe SAs, combined with rapid boronic-ester reactions between phenylboronic acid groups and surface polysaccharides, enabled stable loading without compromising bacterial viability. Fe SAs efficiently scavenged excess ROS, alleviating oxidative stress and maintaining mucosal barrier integrity. Bacterial colonization ensured sustained local retention of the artificial enzymes. In mouse models of IBD, BL@B-SA50 significantly reduced inflammatory cytokines, restored epithelial integrity, reshaped microbiota composition, and reversed dysbiosis. Remarkably, the engineered probiotic effectively treated ulcerative colitis (UC) and Crohn’s disease (CD) in mice as well as UC in dogs, greatly accelerating its translational prospects **(**Fig. 4c**)** [142].

### Surface-engineered bacteria for diverse disease therapeutics

Beyond oncology and intestinal diseases, advances in bacterial surface engineering have expanded the application scope to neurological disorders [143, 144], metabolic diseases [105, 145], and vaccine development. Hu et al. developed a DNA vaccine strategy using plasmids encoding vascular endothelial growth factor receptor 2 (VEGFR2) along with antigenic sequences. DNA was condensed into nanoparticles through electrostatic self-assembly with β-cyclodextrin-PEI, and the cationic PEI facilitated electrostatic modification of *Salmonella*. Surface-functionalized *Salmonella* exhibited enhanced phage resistance, improved systemic dissemination, and increased acid tolerance in the gastrointestinal tract. Following oral administration, the DNA vaccine elicited robust tumor-specific T-cell responses and cytokine production. Concurrent inhibition of tumor angiogenesis through VEGFR2 blockade induced extensive tumor necrosis, offering a promising immunotherapeutic avenue for DNA vaccine-based cancer treatment [146].

### Surface-engineered bacteria for bioimaging and diagnostics

Bioimaging technologies enable multiscale, multimodal visualization of biological systems, providing insights into processes ranging from intracellular signaling to tissue-level dynamics. These tools facilitate early lesion detection, real-time monitoring of disease progression, evaluation of therapeutic response, and guidance for precision interventions. As such, bioimaging underpins individualized treatment strategies, validation of emerging therapeutics, and discovery of non-invasive biomarkers.

With ongoing advances in bacterial surface engineering, engineered bacteria have shown considerable promise in bioimaging, diagnostics, and theranostics. Capitalizing on their intrinsic tropism toward pathological sites (such as tumors and abscesses), imaging agents, including near-infrared dyes and magnetic nanoparticles, can be conjugated onto bacterial surfaces via carbodiimide chemistry (EDC/NHS-mediated coupling of carboxyl and amino groups). After intravenous administration, these conjugates actively home to tumor regions through systemic circulation, enabling high-precision imaging while simultaneously offering therapeutic potential [130].

## Combination therapeutic strategies

The therapeutic efficacy of bacterial therapy derives from the intrinsic tumor-targeting capability of bacteria, their selective colonization and proliferation in the hypoxic cores of solid tumors, and their endogenous toxins as well as their capacity to activate host immunity. Nevertheless, bacterial monotherapy is often insufficient for complete tumor eradication and remains limited by its inherent therapeutic ceiling. Surface engineering enables the conjugation of various therapeutic agents, such as photosensitizers, small-molecule drugs, proteins, and immunomodulators, onto bacterial surfaces. This strategy integrates bacterial therapy with physical, chemical, and immunological treatment modalities, thereby enhancing synergistic antitumor responses and aiming to achieve more effective tumor control and elimination.

### Synergizing surface-modified bacteria with physical therapy for cancer

**Fig. 5:**
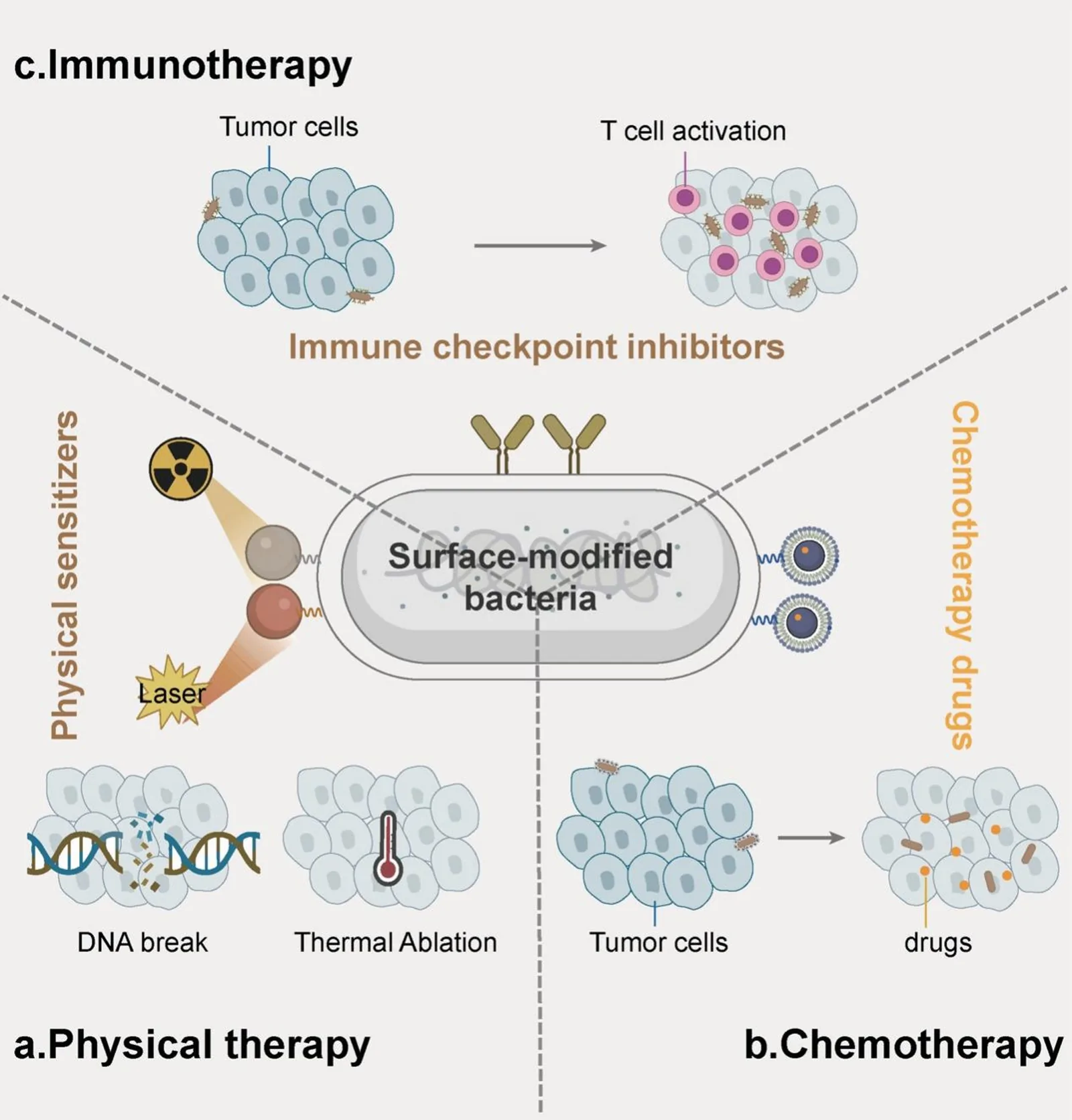
Schematic illustration of surface-modified bacteria as a versatile platform for multimodal cancer therapy. This schematic depicts the integration of surface-engineered bacteria with multiple therapeutic modalities to achieve synergistic antitumor effects. (**a**) Synergizing surface-modified bacteria with physical therapy. Surface-modified bacteria can be functionalized with radiosensitizer or photothermal sensitizer to enable physical ablation of tumor cells. Upon exposure to external stimuli such as X-ray or near-infrared irradiation, these bacteria-mediated physical therapies induce localized DNA damage or thermal ablation, leading to direct tumor cell destruction. (**b**) Synergizing surface-modified bacteria with chemotherapy. Bacteria engineered to carry and deliver chemotherapeutic drugs enable targeted drug accumulation within the tumor microenvironment. The surface conjugation of chemotherapy-loaded nanoparticles or drug molecules facilitates localized DNA damage and tumor cell killing, while minimizing systemic toxicity associated with conventional chemotherapy. (**c**) Synergizing surface-modified bacteria with immunotherapy. Surface modification of bacteria with immune checkpoint inhibitors enables targeted delivery of these immunomodulatory agents to the tumor microenvironment. This strategy potentiates T cell-mediated tumor killing by blocking immune checkpoint pathways locally, thereby amplifying the efficacy of cancer immunotherapy and promoting durable systemic antitumor immunity

Physical therapies, including photothermal therapy (PTT), photodynamic therapy (PDT), and radiotherapy (RT), benefit substantially from the propensity of bacteria to penetrate and accumulate within the hypoxic tumor core. By harnessing this property, photosensitive or radiosensitizing materials can be precisely delivered to tumor sites and activated by external stimuli, enabling potent, localized therapeutic responses. In the context of PTT, Chen et al. conjugated indocyanine green–loaded nanoparticles (INPs) onto the surface of the *Salmonella* strain YB1 via amide bonding for the treatment of subcutaneous bladder cancer. INPs exhibited excellent biocompatibility and efficient photothermal conversion. Notably, YB1 substantially enhanced INP penetration and retention within the hypoxic tumor niche. Upon initial near-infrared (NIR) irradiation, INPs induced focal tumor necrosis and promoted chemotactic bacterial infiltration, resulting in a 14-fold increase in intratumoral YB1 accumulation. Subsequent irradiation elevated local tumor temperatures to 63 °C, enabling the synergistic elimination of large tumors (≥ 500 mm³) and bacteria while achieving remarkable PTT efficacy [147] **(**Fig. 5a**)**. For PDT, the hypoxic tumor microenvironment often restricts ROS production. To overcome this limitation, Liu et al. exploited the oxygen-generating ability of the photosynthetic bacterium *Synechococcus* 7942 (Syne) by conjugating indocyanine green–loaded human serum albumin nanoparticles (HSA/ICG) to the bacterial surface. The combined Syne–HSA system facilitated both active and passive tumor targeting. Under 660 nm irradiation, this platform enabled robust ROS-mediated photodynamic effects while simultaneously alleviating tumor hypoxia via in situ oxygen production, thereby sustaining enhanced PDT-driven immune activation and antitumor responses [148]. For RT enhancement, cytolysin A (ClyA) induces cell-cycle arrest at the radiosensitive G2/M phase, while Bi2S3 nanoparticles (BNPs) act as high–atomic number radiosensitizers released through MMP-2–mediated cleavage within the tumor microenvironment. Leveraging this dual-radiosensitization strategy, Pan et al. engineered *Escherichia coli* MG1655 (eBac) to overexpress ClyA while functionalizing its surface with BNPs via amide conjugation. Following selective tumor colonization, this integrated platform markedly enhanced tumor sensitivity to X-ray irradiation, enabling potent ROS generation and DNA damage at low radiation doses and significantly suppressing tumor growth while minimizing off-target radiotoxicity [149] **(**Fig. 5a**)**.

### Synergizing surface-modified bacteria with chemotherapy for cancer

Chemotherapy relies on chemical agents to eliminate cancer cells and suppress tumor progression. Commonly used drugs—such as camptothecin, doxorubicin (DOX), colchicine, paclitaxel, cisplatin, and carboplatin—are often limited by systemic toxicity and insufficient penetration into distal tumor regions. Poor vascularization and heterogeneous tumor architecture further restrict nanodrug delivery, leading to suboptimal efficacy and high off-target effects. For example, paclitaxel and DOX administered systemically can cause vomiting, alopecia, and cardiotoxicity.

Nanoparticle-based formulations that preferentially accumulate within tumors can enhance local drug concentrations while reducing systemic side effects. Bacteria-mediated nanochemotherapy, benefiting from bacterial tumor tropism and controllable release, significantly improves chemotherapeutic precision and efficacy. For instance, Nguyen et al. decorated paclitaxel-loaded liposomes onto *Salmonella typhimurium* via biotin–streptavidin interactions. Co-culture with NIH/3T3 fibroblasts and 4T1 cancer cells demonstrated superior tumor-cell targeting and cytotoxicity compared to conventional drug-loaded liposomes [118].Xie et al. conjugated DOX to EcN using acid-labile succinic anhydride linkers. EcN’s facultative anaerobic nature enabled tumor-specific accumulation, where acidic conditions triggered linker hydrolysis and controlled DOX release [150]. This strategy enhanced intratumoral drug deposition, improved antitumor efficacy, and reduced systemic toxicity. To further improve drug internalization, DOX and α-tocopheryl succinate (TOS) were conjugated with PEG to form amphiphilic polymers, which were attached to bacterial surfaces through acid-labile click chemistry. Upon bacterial-mediated tumor delivery, these amphiphiles dissociated under acidic conditions and self-assembled into micelles, thereby drastically improving drug internalization into the target cancer cells [151] **(**Fig. 5b**)**.

### Synergizing surface-modified bacteria with immunotherapy for cancer

Combining bacterial therapy with immune checkpoint inhibitors (ICIs) is a widely explored immunotherapeutic approach. While bacterial therapy can reprogram the tumor microenvironment and enhance immune cell infiltration and activation, immunosuppressive signals within tumors often dampen therapeutic efficacy. ICIs relieve T-cell inhibition, thereby synergizing with bacteria-induced antitumor immunity [10].Wang et al. demonstrated that dual surface and intracellular bacterial modification can impart multifunctional therapeutic capabilities. *Escherichia coli* BL21(DE3) was engineered with plasmid pET28a-Mel to intracellularly express photothermal melanin, while αPD-1 antibodies were conjugated to the bacterial surface via in situ dopamine polymerization. This platform enabled uniform and sustained intratumoral co-distribution of both melanin and ICIs. Upon laser irradiation, melanin provided repeated, moderate, and homogeneous heating, potentiating photothermally enhanced immune activation. The co-localization of melanin and αPD-1 further promoted dual immune stimulation, synergistically remodeling the tumor immune microenvironment [152] **(**Fig. 5c**)**.

Beyond combination with endogenous ICIs, bacterial therapy has expanded toward orchestrating coordinated activity among multiple immune cell types. On one hand, surface-engineered bacteria can exploit host immune cells to achieve “secondary targeting” and local immune remodeling. Mi et al. modified *Salmonella* with PEGylated sialic acid–coated silver nanoparticles (SP-AgNPs) capable of recognizing neutrophils. This enabled the bacteria to recruit neutrophils into tumors and, by leveraging their strong infiltration capability, amplify bacterial and nanoparticle accumulation. SP-AgNP, mediated neutrophil depletion, combined with intrinsic cytotoxicity, expanded tumor necrotic regions and further promoted deeper bacterial penetration, achieving synergistic tumor ablation [153].

On the other hand, more advanced strategies utilize bacteria to guide adoptively transferred immune cells into solid tumors. VINCENT et al. injected engineered probiotics capable of releasing synthetic antigens into tumors, establishing a tumor-specific spatial cue. Subsequently infused CAR-T cells were precisely recruited and activated, overcoming barriers of poor infiltration and limited targeting in solid tumors. This “bacteria-guided + CAR-T” approach thus represents a powerful and highly synergistic immunotherapeutic paradigm for solid tumors [154].

## Conclusions and outlook

The clinical experience with first-generation engineered bacteria, exemplified by the VNP20009 trial, has laid bare a fundamental truth: preclinical efficacy does not guarantee clinical response, and the primary barrier lies not in the potency of the bacterial cargo, but rather in the failure of systemically delivered bacteria to achieve sufficient accumulation at pathological sites. As we have defined in this Review, this concept of “tumor accessibility”, the ability to survive immune surveillance, home to target tissues, and penetrate the disease microenvironment, represents the sine qua non for therapeutic efficacy. Inadequate tumor accessibility is the root cause of clinical failure in bacterial therapy. When accessibility is compromised, downstream consequences follow. First, poor accessibility leads to the clearance of the majority of administered bacteria during circulation, exposing the surviving population to sustained immune clearance and oxidative stress, which compromises viability and risks genetic instability [50]. Second, the reduced number of bacteria reaching the tumor necessitates high-dose infusions in clinical settings (Toso et al., David et al.), which elevates endotoxin exposure. Given humans are far more endotoxin-sensitive than mice [155, 156], this triggers severe inflammation, dose-limiting toxicities (DLTs), and a narrowed therapeutic window. Third, because bacterial toxicity is dose-dependent, the need for attenuation to manage DLTs often leads to over-attenuation, which further diminishes colonization fitness and therapeutic efficacy [157, 158]. Together, these effects restrict the therapeutic window, making poor accessibility the core translational barrier. Addressing this clinical bottleneck requires a paradigm shift in how we approach bacterial engineering. Surface modification, as surveyed in this Review, offers a direct route to enhance each determinant of accessibility: evading immune clearance, enhancing colonization at disease sites, and facilitating tissue penetration.

Bacterial surface engineering is entering a critical transition from method-driven innovation to function-driven system design. Rather than viewing physical, chemical, and biological modifications as parallel strategies, a more unifying perspective is to consider how these approaches collectively regulate the host–bacteria interface, which ultimately determines in vivo fate, therapeutic efficacy, and safety boundaries. In this context, current strategies are not merely limited by individual technical drawbacks, but by an incomplete ability to precisely control the spatiotemporal interactions between engineered bacteria and complex biological environments. A central challenge that emerges is the lack of dynamic interfacial controllability. Physical coatings, while generally biocompatible, often fail under physiological shear forces and enzymatic remodeling, leading to rapid loss of function. Importantly, bacterial proliferation further exacerbates this limitation: as bacteria undergo growth and division, the expansion and remodeling of the cell envelope can disrupt or shed surface-bound coatings, resulting in progressive loss of structural integrity and increased heterogeneity in functional display. This proliferation-associated destabilization introduces an additional layer of unpredictability in vivo, complicating precise control over therapeutic activity. Chemical modifications improve functional density and modularity but remain constrained by static conjugation and limited responsiveness [159]. Biological engineering introduces programmability, yet its reliance on genetic circuits brings intrinsic trade-offs between functional complexity, metabolic burden, and biosafety risks, including horizontal gene transfer and ecological persistence. These limitations collectively highlight that current designs largely operate as static or semi-dynamic systems in inherently dynamic in vivo environments.

Looking ahead, the evolution of bacterial surface engineering will converge on multifunctionality, controllability, and clinical-grade standardization—all oriented toward the ultimate goal of ensuring systemic accessibility in human patients. To move the concept of systemic accessibility from qualitative description toward actionable guidance, quantitative benchmarks and a multidimensional evaluation framework are urgently needed. Two specific points merit emphasis. First, initial clinical quantification has already emerged: a human trial defined > 10⁷ CFU/g as “high level of bacterial colonization”, providing a rare and valuable human-derived reference point [29]. Although not yet a consensus standard, this threshold indicates that the field is beginning to adopt quantitative thinking. Second, future assessments of tumor accessibility should integrate at least three complementary dimensions: circulatory half-life (reflecting immune evasion and tumor-homing capacity), intratumoral colonization titer (reflecting colonization density), and tumor infiltration depth (reflecting physical penetration capacity). The precise numerical thresholds for these parameters will require refinement through future clinical-correlative studies; nevertheless, establishing this multidimensional framework represents a necessary first step toward standardization.

Future systems must transcend single-function coatings and instead integrate specific recognition and controllable activation modules capable of adapting to tumor heterogeneity and dynamic immune states. To ensure clinical manageability, built-in safety switches (e.g., inducible expression switches [160–163], kill switches [164, 165] or auxotrophic mutations [166, 167] are essential for precise and safe therapy. Moreover, the integration of molecular imaging, machine learning-guided design, and patient-specific profiling may enable bacteria that operate as intelligent, self-adjusting therapeutic entities. Preclinical validation of these advanced systems must shift from standard subcutaneous xenografts to clinically relevant models, such as patient-derived xenografts or orthotopic models, that better recapitulate the human tumor microenvironment and its associated barriers to bacterial accessibility [168, 169]. Formulation strategies must be route-specific, intravenous formulations should prioritize circulation half-life and biocompatibility [170], while oral formulations require protection against gastrointestinal degradation and enhancement of intestinal epithelial translocation [171]. At the translational level, scalable Good Manufacturing Practice (GMP) is esstential, yet live bacterial products introduce unique manufacturing complexities—including plasmid instability, strain drift, and batch‑to‑batch viability variability, all of which demand stringent process controls [50]. These challenges are compounded by the lack of comprehensive, product‑specific GMP guidelines for live bacterial therapeutics, a regulatory gap that must be urgently addressed to facilitate clinical translation.

Overall, bacterial surface engineering is evolving toward a new paradigm centered on interface programmability and systems-level integration. The next generation of engineered bacteria will not be defined solely by enhanced targeting or payload delivery, but by their ability to achieve systemic accessibility, to reliably reach, infiltrate, and function within target lesions in the human body—while operating as self-regulating, clinically controllable living systems.

## Data Availability

No datasets were generated or analysed during the current study.

## Abbreviations

ADC: Antibody‑drug conjugate
XDC: Drug conjugate
CAR: Chimeric antigen receptor
BMBL: Biosafety in Microbiological and Biomedical Laboratories
BSL: Biosafety level
MAC: Membrane attack complex
CD: Cytosine deaminase
5-FC: 5-fluorocytosine
5-FU: 5‑fluorouracil
BMDM: Bone marrow-derived macrophage
MHC-II: Major histocompatibility complex II
TAM: Tumor-associated macrophages
NHS: N‑hydroxysuccinimide
EDC: 1-ethyl-3-(3-dimethylaminopropyl)carbodiimide
PDA: Polydopamine
DBCO: Dibenzocyclooctyne
PG: Peptidoglycans
LPS: Lipopolysaccharide
Kdo: 3-deoxy-D-manno-oct-2-ulosonic acid
ncAAs: Noncanonical amino acids
RGD: Arginine-glycine-aspartate
INP: Ice nucleation protein
EPS: Extracellular polysaccharides
PAMP: Pathogen-associated molecular pattern
SCV: Salmonella-containing vacuole
PLGA: Poly(lactic-co-glycolic acid)
IBD: Inflammatory bowel disease
ROS: Reactive oxygen species
UC: Ulcerative colitis
VEGFR: Vascular endothelial growth factor receptor
PTT: Photothermal therapy
PDT: Photodynamic therapy
RT: Radiotherapy
DOX: Doxorubicin
ICI: Immune checkpoint inhibitor
DLT: Dose-limiting toxicity
GMP: Good Manufacturing Practice
